# Engineering Orthogonal Methyltransferases to Create Alternative Bioalkylation Pathways

**DOI:** 10.1002/anie.202004963

**Published:** 2020-06-22

**Authors:** Abigail J. Herbert, Sarah A. Shepherd, Victoria A. Cronin, Matthew R. Bennett, Rehana Sung, Jason Micklefield

**Affiliations:** ^1^ Department of Chemistry and Manchester Institute of Biotechnology The University of Manchester 131 Princess Street Manchester M1 7DN UK

**Keywords:** bioalkylation, biotransformations, carboxymethylation, enzyme cofactors, methyltransferases

## Abstract

*S*‐adenosyl‐l‐methionine (SAM)‐dependent methyltransferases (MTs) catalyse the methylation of a vast array of small metabolites and biomacromolecules. Recently, rare carboxymethylation pathways have been discovered, including carboxymethyltransferase enzymes that utilise a carboxy‐SAM (cxSAM) cofactor generated from SAM by a cxSAM synthase (CmoA). We show how MT enzymes can utilise cxSAM to catalyse carboxymethylation of tetrahydroisoquinoline (THIQ) and catechol substrates. Site‐directed mutagenesis was used to create orthogonal MTs possessing improved catalytic activity and selectivity for cxSAM, with subsequent coupling to CmoA resulting in more efficient and selective carboxymethylation. An enzymatic approach was also developed to generate a previously undescribed co‐factor, carboxy‐*S*‐adenosyl‐l‐ethionine (cxSAE), thereby enabling the stereoselective transfer of a chiral 1‐carboxyethyl group to the substrate.

## Graphical abstract

**Methyltransferase enzymes** (MTs) selectively deliver methyl substituents to an array of molecules, thereby controlling gene expression or modulating the bioactivity of therapeutically important natural products. A MT was engineered for selective carboxy(m)ethylation to give an orthogonal MT that was combined with the SAM‐derivatising enzyme CmoA to create new carboxymethylation pathways.

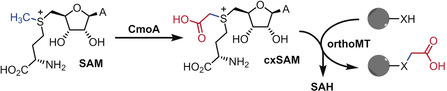

## Introduction

Methylation is one of the simplest and most widely used reactions in nature. The majority of methylation reactions in cells are catalysed by *S*‐adenosyl‐l‐methionine (SAM)‐dependent methyltransferase (MT) enzymes that transfer a methyl group from SAM to nucleophilic centres in a wide array of substrates, including small metabolites, proteins, nucleic acids, and other biological molecules.^1^, ^2^, ^3^, ^4^, ^5^ Typically, MTs transfer methyl groups with exquisite chemo‐ and regio‐selectivity, which often has a profound effect on the properties of biological molecules, thus making them a highly diverse and important class of enzymes. For example, methylation of proteins and DNA is a key mechanism in the epigenetic control of gene expression.^1^, ^2^ MT‐catalysed methylation is also a common theme in the biosynthesis of therapeutically important secondary metabolites, including antibiotics, where methyl substituents can affect the properties and bioactivity of the natural products (NPs).^3^, ^5^

**Figure 1:**
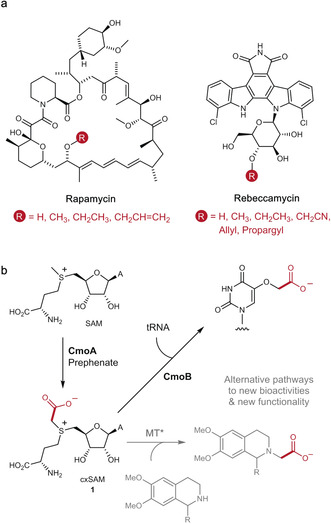
a) Alkyl diversification of natural products: rapamycin and rebeccamycin derivatives generated using MTs and SAM analogues.^11^, ^12^ b) CmoA‐catalysed formation of cxSAM. In the native pathway, cxSAM functions as a co‐factor for the carboxymethyltransferase CmoB in the modification of a tRNA substrate. In this work (grey), we show how CmoA can be combined with an engineered methyltranferase (MT*), thereby opening up new pathways to carboxymethylated products.

Several SAM‐dependent MTs have been shown to accept synthetic SAM analogues, with alternative S‐alkyl, S‐allyl, or S‐propargyl substituents, facilitating non‐native alkylation reactions.^5^, ^6^, ^7^, ^8^, ^9^, ^10^, ^11^, ^12^, ^13^, ^14^, ^15^, ^16^ The ability to transfer functional groups regioselectively using MTs and SAM derivatives has been exploited for the site‐selective labelling of proteins and DNA,^5^, ^6^, ^7^, ^8^, ^9^, ^10^ as well as in the structural diversification of clinically important NPs, including the anticancer agent rebeccamycin and rapamycin immunosuppressive agents (Figure 1 a).^11^, ^13^ In most cases, the SAM analogues are prepared synthetically through the alkylation of *S*‐adenosyl‐l‐homocysteine (SAH); this process however gives rise to a mixture of sulfonium epimers, with the natural *S* epimer functioning as a cofactor and the *R* epimer potentially inhibiting activity. Some SAM analogues can be prepared from synthetic methionine derivatives and ATP using a methionine adenosyltransferase (MAT) enzyme to produce the biologically relevant sulfonium epimer as the sole product.^11^, ^13^ MAT has also been used to produce related seleno‐SAM analogues.^14^, ^15^ Notwithstanding this, SAM analogues are expensive to produce, unstable, and cannot penetrate the cell membrane, thus largely limiting their utility to small scale in vitro applications.^16^, ^17^, ^18^, ^19^, ^20^

New enzymatic approaches to produce SAM derivatives from metabolically available, rather than synthetic, precursors would be highly desirable. Such approaches, combined with engineered orthogonal MTs that are selective for enzymatically derived SAM analogues, could open up new bioalkylation pathways in vitro and in vivo. To this end, we were particularly interested in the discovery of a naturally occurring SAM derivative, carboxy‐*S*‐adenosyl‐l‐methionine (cxSAM), which is generated from SAM and prephenate by the cxSAM synthase (CmoA) enzyme (Figure 1 b).^21^, ^22^ CmoA is found in bacterial species and functions in tandem with a carboxymethyl transferase (CmoB), which transfers the carboxymethyl group from cxSAM to a tRNA substrate.^23^ Whilst another pathway was recently discovered that utilises cxSAM in the carboxymethylation of a peptide substrate,^24^ such bioalkylation pathways are extremely rare in nature. However, if CmoA could be coupled with orthogonal variants of the more common MTs, engineered to have higher selectivity for cxSAM, then a large array of diverse carboxymethylated products could be generated with new functionality and bioactivity. In this paper, we demonstrate how CmoA can be utilised in tandem with MT enzymes to generate carboxymethylated, rather than methylated, products. Furthermore, we demonstrate how active‐site mutagenesis can lead to orthogonal MTs with improved selectivity for cxSAM over SAM, thereby opening the way to engineering new carboxymethylation pathways in vitro and in vivo. Finally, we show how CmoA can also be used to generate a novel carboxy‐*S*‐adenosyl‐l‐ethionine (cxSAE) cofactor, thereby facilitating the stereoselective MT‐catalysed transfer of a chiral 1‐carboxyethyl group.

## Results and Discussion

### Activity of Methyltransferases (COMT & CNMT) with Synthetic cxSAM.

**Figure 2:**
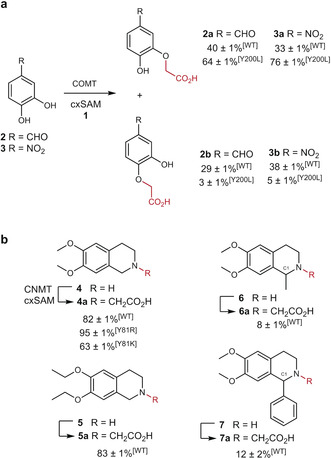
a) Catechol carboxymethylation assays with synthetic cxSAM, catechols **2** or **3** (0.25 mm), COMT (100 μm), MgCl_2_ (6 mm), and DTT (1 mm), incubated for 20 h. b) THIQ carboxymethylation assays. CNMT was pre‐incubated with THIQ **4** or **5** for 1 h to remove co‐purified SAM and then transferred to fresh buffer. Assays were then conducted with synthetic cxSAM (1.6 mm), substrate **4**–**7** (0.25 mm) and CNMT (100 μm) and incubated for 17 h. Assays were done in triplicate and standard errors calculated.

To assess the possibility of using cxSAM as an alternative cofactor to SAM, we chose to explore reactions catalysed by catechol‐O‐methyltransferase (COMT) from *Rattus norvegicus* and coclaurine‐N‐methyltransferase (CNMT) from *Coptis japonica*. COMT was previously shown to accept a range of catechol substrates as well as synthetic SAM analogues.^25^ Accordingly, WT COMT was incubated with 3,4‐dihydroxybenzaldehyde **2** and synthetic cxSAM, prepared through alkylation of SAH with bromoacetic acid,^21^ resulting in a mixture of *meta*‐ and *para*‐carboxymethylated regioisomers **2 a** (40 %) and **2 b** (29 %), respectively (Figure 2 a and Figure S1 in the Supporting Information). These results are consistent with the relaxed regioselectivity observed for methylation reactions catalysed by COMT.^25^ Previously we showed that a COMT Y200L mutant has significantly improved regioselectivity, affording predominately *meta*‐methylation of various substituted catechol derivatives.^25^ In light of this, catechol **2** and cxSAM were similarly incubated with COMT Y200L, affording significantly improved regioselectivity for *meta*‐ over *para*‐carboxymethylation (**2 a** 64 % vs. **2 b** 3 %). Similar results were obtained for 4‐nitrocatechol **3** and cxSAM, with COMT Y200L providing 76 % *meta*‐ and 5 % *para*‐carboxymethyl products **3 a** and **3 b**, respectively (Figure 2 a). For both catechol substrates **2** and **3**, small amounts of the corresponding methylated products were observed (e.g., **2** with WT and Y200L COMT gave 21±1 % and 16±1 % methylated products, respectively), which is attributed to co‐purification of SAM with COMT and background decarboxylation of cxSAM to SAM, which is apparent during the incubation period.

In our previous studies, we also showed that CNMT accepts a variety of tetrahydroisoquinoline (THIQ) substrates as well as SAM analogues.^26^ To further explore its cofactor selectivity, WT CNMT was incubated with synthetic cxSAM and various THIQ substrates (**4**–**7**; Figure 2 b and Figure S2). THIQs **4** and **5** showed significant carboxymethylation (82 and 83 % respectively), whilst THIQs **6** and **7** gave only low conversions with cxSAM (8–12 %). Using the crystal structure of CNMT (PDB ID: 6GKV),^26^ we infer that the close proximity of the carboxymethyl group of cxSAM and the C1‐substituent of the THIQs (**6** and **7**) is likely to cause steric hindrance leading to decreased activity.

### Site‐Directed Mutagenesis of CNMT.

**Figure 3:**
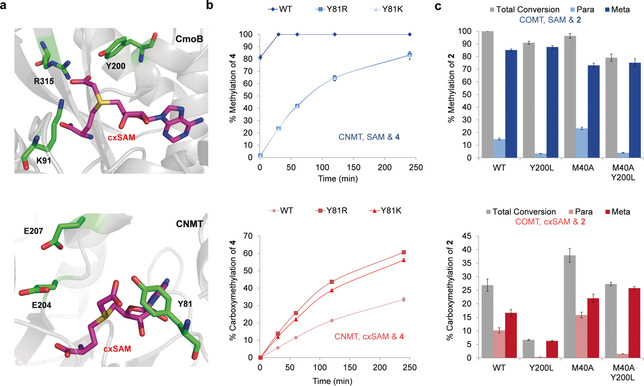
a) Crystal structure of CmoB and a model of cxSAM binding into the CNMT active site. Top: The CmoB active site highlights three residues thought to interact with the carboxymethyl group (PDB ID: 4QNU). Bottom: WT CNMT with cxSAM positioned in the active site (based on PDB ID: 6GKV, which has SAH bound), highlighting three residues predicted to be in proximity to the carboxymethyl group. b) CNMT time courses with THIQ **4** (0.25 mm) and SAM or cxSAM were conducted using either 1 mm SAM (top/blue) or 1.6 mm cxSAM (bottom/red), with commercial SAM comprising only the active *S* enantiomer at 80 % purity and cxSAM being synthesised as a mixture of enantiomers. c) COMT activity assays with catechol **2** (0.2 mm) and SAM or cxSAM were conducted for 5 h at 37 °C to compare % methylation and carboxymethylation, using either 1 mm SAM (top/blue) or 1.6 mm cxSAM (bottom/red). Assays were done in triplicate and standard errors calculated.

Having established that CNMT can utilise cxSAM as a cofactor, we sought to develop CNMT mutants with increased activity and selectivity for cxSAM, since SAM will ultimately be present in significant levels within tandem CmoA‐CNMT cascade reactions. CmoB, which naturally functions in tandem with CmoA, exhibits approximately 500‐fold higher affinity for cxSAM over SAM, which presumably ensures that no significant competing methylation of its tRNA substrate occurs in vivo.^21^, ^23^ In light of this, we used the reported X‐ray crystal structure of CmoB (PDB ID: 4QNU) to guide mutagenesis of CNMT.^26^ Due to the low sequence similarity between CNMT and CmoB (17.5 % identity matrix, CLUSTAL 2.1), only those residues in close proximity to the cofactor were considered. Within the CmoB active site, Lys91, Tyr200, and Arg315 residues were identified, which form interactions with the S‐carboxymethyl group of cxSAM, likely contributing to the high selectivity of CmoB (Figure 3 a).^23^ A model of CNMT in complex with cxSAM, which possesses a natural *S*‐configured sulfonium centre, indicates that Glu204, Glu207, and Tyr81 residues would be in closest proximity to the S‐carboxymethyl group. These residues were each subjected to site‐directed mutagenesis, introducing either smaller residues that could provide more space to accommodate the larger carboxymethyl group, or basic residues which may form favourable electrostatic (salt‐bridge) interactions with the carboxylic acid moiety.

Eleven CNMT mutants in total (E204A/S/K, E207G/S/K/R, and Y81F/H/K/R,) were assessed for activity with THIQ **4** and cxSAM. In order to reduce competing methylation occurring due to co‐purification of SAM with CNMT, the enzymes were pre‐incubated for an hour with THIQ **5** and washed with reaction buffer prior to assay with cxSAM and **4**. All E204 and E207 mutants tested showed complete loss of activity with cxSAM. The E204 residue is situated close to the sulfonium centre of the cofactor and ammonium ion of the substrate, which suggests it may be important for substrate and/or cofactor binding via electrostatic interactions.^26^ In contrast, all of the Y81 mutants showed comparable or higher activity with cxSAM and significantly lower activity with SAM compared with the WT, with the Y81R mutant showing greatest cxSAM selectivity (Figures S3, S4). At this stage, we have not pursued determination of accurate kinetic parameters because the synthetic cxSAM produced is a mixture of diastereoisomers, the separation of which is problematic due to the instability of the cofactor. In addition, as stated above, CNMT co‐purifies with SAM, which would further complicate determination of accurate kinetic parameters. We instead looked to demonstrate enzyme selectivity through separate time course assays for WT and Y81R/K CNMT with SAM or cxSAM and substrate **4** (Figure 3 b). This showed that methylation of **4** with SAM by the WT CNMT is complete in 30 min, whilst both Y81R/K mutants afford less than 25 % methylation over the same time period. In contrast, carboxymethylation of **4** with cxSAM is 23 % and 28 % higher than the WT for Y81K and Y81R, respectively, across the 0–4 h time period. The observation that Y81K affords lower levels of carboxymethylated product (**4 a**) compared with the WT in 17 h assays described above (Figure 2 a) may be due to instability of the Y81K mutant over the longer time course.

In addition, competition assays, with varying ratios of SAM/cxSAM and THIQ substrate **4**, also reveal that mutants Y81R/K have improved selectivity for cxSAM (Figure S5). We hypothesise that the basic side chains of R/K81 mutants may be suitably positioned to form polar interactions with the carboxyl group of cxSAM similar to the interactions observed in CmoB.^19^, ^21^ Taken together, the data presented here provides a proof‐of‐concept that the selectivity of MTs for cxSAM can be significantly improved by rational targeted mutagenesis.

### Site‐Directed Mutagenesis of COMT.

A similar approach was adopted to engineer COMT variants with higher selectivity for cxSAM. Within the active site of COMT, we observed a methionine residue (M40) in close proximity (2.7 Å) to the methyl group of SAM (Figure S6). Accordingly, six point mutants were produced (M40K/R/H/A/S/C) in order to alter the electrostatic and steric interactions of this residue with cxSAM. Of these mutants, M40A showed the most significant increase in activity with cxSAM (Figure 3 c and Figure S7). We propose that this is due to increased space surrounding the carboxymethyl group of cxSAM within the active site, leading to reduced steric hindrance. Whilst M40A has increased activity with cxSAM, the regioselectivity of carboxymethylation was poor. Since the COMT Y200L mutant is known to achieve a high regioisomeric excess (*re*) of more than 90 % *meta*,^25^ we produced an M40A/Y200L double mutant. Assays conducted over five hours showed that the double mutant has increased regioselectivity with cxSAM, affording *meta*‐carboxymethylation with high *re* (94 %; Figure 3 c). Moreover, M40A/Y200L also showed a 21 % decrease in methylation yields when assayed with SAM, compared to the WT. Although both the M40A and M40A/Y200L mutants exhibited improved selectivity for cxSAM versus SAM, further mutagenesis may be necessary to deliver COMT variants with higher selectivity for more efficient in vitro or in vivo carboxymethylation reactions.

### CmoA‐MT Coupled Assay.

**Figure 4:**
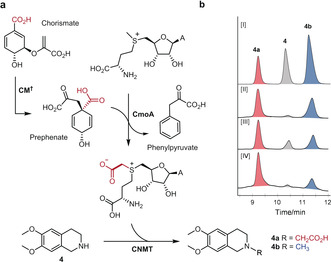
a) Enzymatic cascade reaction for in vitro THIQ carboxymethylation. CM^†^ can be omitted from the reaction since the rearrangement of chorismate to prephenate can proceed spontaneously. b) HPLC traces of cascade reactions. [I] mixed standards; [II] assay of WT CNMT and CmoA without optimisation; [III] assay of CNMT Y81R and CmoA without optimisation; [IV] assay of CNMT Y81R and CmoA following optimisation as shown in Figure S10 E. HPLC peaks: grey=starting material **4**; red=carboxymethylation product **4 a**; blue=methylation side product **4 b**.

We next sought to couple CmoA with CNMT to generate cxSAM enzymatically for carboxymethylation of the THIQ substrate **4** in a cascade reaction (Figure 4 a). Initially, we envisaged using chorismate mutase (CM) to generate prephenate from chorismate, which can be isolated in high yield from an *E. coli* KA12 CM‐deletion strain.^27^, ^28^ However, after optimising conditions for the production of cxSAM from SAM and chorismate with CM and CmoA, it was apparent that equivalent and in some cases higher yields of cxSAM could be generated if CM was omitted from the reaction (Figures S8, 9). Chorismate is well known to undergo a spontaneous Claisen rearrangement to prephenate, and although CM accelerates this reaction a million‐fold, the rate for the non‐enzymatic rearrangement is sufficient for efficient CmoA‐catalysed SAM carboxylation. Following optimisation, a tandem coupled assay was developed that utilises chorismate and CmoA to give cxSAM (ca. 80 %), with some residual SAM (ca. 20 %) remaining, followed by addition of CNMT and the substrate **4**. Through this method, CmoA and the more cxSAM‐selective CNMT Y81R mutant afforded 70±1 % conversion of THIQ **4** to carboxymethylated product **4 a**, with only 16±1 % methylated THIQ **4 b** produced (Figure 4 b, Figures S10, 11). Whilst not completely selective, the final 4.5:1 ratio of carboxymethylation to methylation observed with CmoA‐CNMT (Y81R) compares favourably with the coupled assays with CmoA‐WT CNMT, which give a 1:1 mixture of carboxymethylated to methylated products. Since both SAM and prephenate are present in bacterial cells, we envisage that the combination of CmoA and an engineered MT, with higher selectivity for cxSAM over SAM, may have potential to generate carboxymethylated products in vivo as well as in vitro.

### Carboxy‐*S*‐adenosyl‐l‐ethionine (cxSAE) and CNMT‐Catalysed Carboxyethylation.

**Figure 5:**
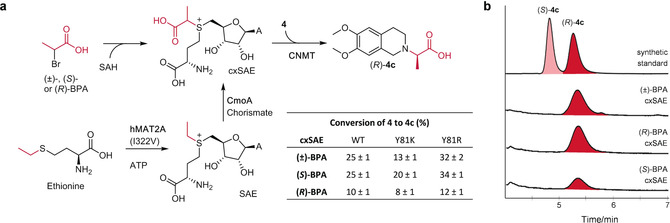
a) Routes to in vitro carboxyethylation. Synthesis of cxSAE from SAH and BPA, or enzymatic production of cxSAE using hMAT and CmoA, followed by subsequent CNMT‐catalysed carboxyethylation of THIQ **4**. The table insert shows C18 HPLC conversions of THIQ **4** to (*R*)‐**4 c** when assayed with WT, Y81R, and Y81K CNMT enzymes and synthetic cxSAE generated from (±)‐, (*S*)‐, or (*R*)‐BPA. Assays were done in triplicate and standard errors calculated. b) Independent chiral HPLC assay analysis of carboxyethylated heliamine products from CNMT Y81K and (±)‐, (*S*)‐, or (*R*)‐BPA‐derived synthetic cxSAE.

In order to further expand the synthetic utility of CmoA‐MT cascade reactions, we envisaged generating the new cofactor carboxy‐*S*‐adenosyl‐l‐ethionine (cxSAE), which may enable MT‐mediated transfer of a chiral 1‐carboxyethyl group (Figure 5 a). To test this, synthetic cxSAE, generated from alkylation of SAH with (±)‐2‐bromopropionic acid (BPA), was incubated with CNMT WT as well as the Y81K and Y81R mutant enzymes (Figure S12). The WT, Y81K, and Y81R enzymes showed significant activity with synthetic cxSAE, giving 25, 13, and 32 % of 1‐carboxyethyl THIQ **4 c**, respectively. To determine the configuration of the product **4 c**, the substrate THIQ **4** was also separately alkylated with (*S*)‐ and (*R*)‐BPA. However, chiral HPLC analysis revealed that the alkylation of **4** proceeds with significant racemisation. This is likely due to enolisation of BPA at the elevated temperature of the reaction (>40 °C).^29^ In addition, BPA can form an α‐lactone, which could lead to the alkylation of **4** proceeding with overall retention of configuration, whilst direct alkylation with BPA proceeds with inversion of stereochemistry. In light of this, **4** was alkylated with (*R*)‐ and (*S*)‐BPA methyl esters and then hydrolysed to afford 1‐carboxyethyl THIQ standards, (*S*)‐**4 c** and (*R*)‐**4 c**, in high enantiomeric excesses (*ee*; 95 % *ee* in each case). Using these standards, it was apparent from chiral HPLC that CNMT‐catalysed carboxyethylation of THIQ **4** with synthetic cxSAE, generated from racemic BPA, is completely stereoselective giving only (*R*)‐**4 c**. To further probe the stereochemistry of this process, synthesis of cxSAE was also attempted using homochiral BPA methyl esters, however alkylation reactions in water or mixed aqueous/organic solvents proved problematic. Therefore, SAH was separately alkylated with (*R*)‐ and (*S*)‐BPA at low temperature to minimise racemisation, and the resulting cxSAEs were similarly incubated with WT, Y81K, and Y81R CNMT and THIQ substrate **4**. Whilst the yields of 1‐carboxyethyl THIQ were similar for (±)‐ and (*S*)‐BPA‐derived cxSAE, the yields with (*R*)‐BPA‐derived cxSAE were significantly reduced. Surprisingly, however, the *R*‐configured carboxyethyl THIQ product (*R*)‐**4 c** was formed in all cases (Figure 5 b).

Four diastereoisomers of cxSAE can be produced upon alkylation of SAH with BPA. However, the separation of these diastereoisomers and subsequent determination of their absolute configurations is very difficult, particularly given their instability. Consequently, it was not possible to reach any conclusions about the stereochemical course of the process using synthetic cxSAE. To avoid the complication of using diastereoisomeric mixtures of cxSAE, we attempted to generate cxSAE enzymatically, which should afford only one stereoisomer of cxSAE. Firstly, a human methionine adenosyltransferase (hMAT2A) mutant (I322V), which is known to show promiscuity with methionine derivatives,^11^, ^17^ was used to form *S*‐adenosyl‐l‐ethionine (SAE) from ATP and ethionine (Figure 5 a). CmoA was then added along with prephenate to form cxSAE in 13 % yield. The enzymatic cxSAE was then incubated with CNMT and THIQ **4**, leading to production of carboxyethyl THIQ **4 c**. As with the synthetic cxSAE, only (*R*)‐**4 c** was evident from chiral HPLC–MS (Figure S13). Based on the crystal structure of CmoA in complex with cxSAM (PDB ID: 4GEK) and computational predictions of likely positions of precursors SAM and prephenate in the CmoA active site,^21^ we predict that cxSAE would possess 1‐(*S*)‐carboxyethyl group and an *S*‐configured sulfonium centre (Figure S14). In light of this, we conclude that the CNMT carboxyethylation of THIQ **4** with cxSAE proceeds with inversion of configuration, which is also consistent with the native methylation reactions of MT, which have been shown to proceed with inversion using SAM possessing a chiral methyl group.^28^ Taken together, these results suggest that CxSAE possessing a 1‐(*R*)‐carboxyethyl group, generated synthetically from BPA, is not turned over by CNMT.

## Conclusion

In summary, we have shown that two typical members of the ubiquitous Class I methyltransferase, COMT and CNMT, can utilise cxSAM as an alternative cofactor to generate carboxymethylated products. Structure‐guided mutagenesis was used to engineer COMT and CNMT mutants with improved selectivity for cxSAM. This enabled a tandem enzyme reaction to be developed with the cxSAM synthase (CmoA) and CNMT, delivering carboxymethyl‐THIQ in good yields. To the best of our knowledge, there are no other reports describing the combination of CmoA with MTs to create new pathways to carboxymethylated rather than methylated products. Previous approaches to produce SAM analogues as co‐factors for MTs have relied on multistep chemical or enzymatic synthesis for in vitro applications, which are limited by the costs of precursor and the instability of SAM analogues. Our approach opens up the possibility of combining CmoA and engineered MTs to create new structural diversity, in vitro and in vivo, from entirely natural precursors, thereby obviating the need for multistep chemical or enzymatic synthesis and purification steps.

MTs are amongst the most common enzymes in nature, methylating a vast array of substrates, from small metabolites to biomacromolecules. Addition of a charged and polar carboxymethyl group, rather than a small hydrophobic methyl substituent, could therefore lead to many new products with significantly altered physiochemical properties, biological activities, and potentially new functions. Moreover, the additional carboxyl group provides a handle for further selective derivatisation using conventional amide coupling chemistry or powerful metallaphotoredox reactions,^31^, ^32^, ^33^, ^34^ that can be performed under mild aqueous (biocompatible) conditions.^35^ Such downstream chemistry could be used to fine‐tune the properties of bioactive molecules and can provide further opportunities for regioselective bioconjugation and labelling.^5^, ^6^, ^7^, ^8^, ^9^, ^10^ Finally, we have demonstrated that CmoA can be used to generate a new co‐factor, cxSAE, which was accepted by CNMT, thereby enabling the stereoselective transfer of a chiral 1‐carboxyethyl group to a THIQ substrate. Several C‐methyltransferases, including GlmT and MppJ,^36^, ^37^, ^38^ have been described that transfer a methyl group to a prochiral substrate and create a new stereogenic centre. However, we are unaware of any other examples where a MT has been shown to catalyse the stereoselective transfer of a chiral functional group from the co‐factor to the substrate.

## Conflict of interest

The authors declare no conflict of interest.

## Supporting information

As a service to our authors and readers, this journal provides supporting information supplied by the authors. Such materials are peer reviewed and may be re‐organized for online delivery, but are not copy‐edited or typeset. Technical support issues arising from supporting information (other than missing files) should be addressed to the authors.

Supplementary
