# Supplementary material for: Engineering Orthogonal Methyltransferases to Create Alternative Bioalkylation Pathways
Source: Angew Chem Int Ed Engl. 2020 Jun 22;59(35):14950–6. doi: 10.1002/anie.202004963 (PMC7496830; doi:10.1002/anie.202004963)
Supplement: Supplementary file 1 — Supplementary [file ANIE-59-14950-s001.pdf]

Angewandte Chemie
Eine Zeitschrift der Gesellschaft Deutscher Chemiker

Supporting Information

Engineering Orthogonal Methyltransferases to Create Alternative Bioalkylation Pathways

*Abigail J. Herbert, Sarah A. Shepherd, Victoria A. Cronin, Matthew R. Bennett, Rehana Sung, and Jason Micklefield**

anie_202004963_sm_miscellaneous_information.pdf

Table of Contents

Experimental Procedures 1
Supplementary Figures 8
Supplementary Tables 32
References 33
Author Contributions 33

Experimental Procedures

Materials. Chemical reagents and solvents were purchased from commercial sources (Sigma Aldrich, Fisher Scientific) and used without further purification unless otherwise stated. Molecular biology enzymes were purchased from New England Biolabs unless otherwise stated. Oligo nucleotides were synthesised by Sigma Aldrich.

Construction of protein expression plasmids. The plasmids pET28a-CNMT, pET21b-COMT, pET21b-COMT Y200L and pNIC28-Bsa4-hMAT2A (I322V) were constructed as we described previously.[5,11,25] The pET28a-CmoA plasmid was created from a copy of the CmoA gene which was amplified from genomic DNA of *E.coli* BL21 (DE3) by PCR using the primers CmoA-F and CmoA-R (see **Table S1**). The amplified CmoA gene was subsequently cloned into a pET28a vector (Novagen), using *NdeI* and *XhoI* restriction enzymes and verified by DNA sequence analysis (GATC Biotech). This vector was used to transform competent *E.coli* DH5α cells, and the desired transformants were selected for kanamycin resistance. The pET28a-CmoA plasmid was isolated and then used to transformed *E. coli* BL21 (DE3) for protein expression. A codon optimised synthetic CM gene was purchased from Genewiz in a pUC57-Kan vector. The sequence of CM was derived from *M. janaschii* (MjCM, PDB: 2GTV) which was modified as described by MacBeath *et al.*[S1] The modified CM gene was subcloned into the pET21b vector using restriction enzymes *NdeI* and *HindIII*. The pET21b-CM construct was verified by DNA sequence analysis (GATC Biotech) and subsequently used to transform competent *E.coli* DH5α cells, and transformants were selected for ampicillin resistance.

Protein expression and purification. *E.coli* BL21 (DE3) transformants (or Rosetta™ 2 in the case of hMAT2A (I322V)) were cultivated in LB media supplemented with either 50 µg mL^{-1} kanamycin (pET28a-CmoA, pET28a-CNMT) or 50 µg mL^{-1} ampicillin (pET21b-COMT, pET21b-CM) at 37 ˚C with constant agitation (220 rpm) until a suitable cell density was reached (OD600 = 0.6). Protein expression was induced by the addition of IPTG (0.5 mM) and cells were further cultivated for 18 h (220 rpm, 18 ˚C), prior to being harvested by centrifugation. Pelleted cells were re-suspended in the appropriate lysis buffer (CmoA: 20 mM HEPES, 150 mM KCl, pH 7.5; COMT: 50 mM potassium phosphate buffer, 300 mM NaCl, pH 7.4; CNMT: 20 mM potassium phosphate buffer, 200 mM NaCl, 20 mM imidazole, pH 7; CM: 10 mM potassium phosphate buffer, 160 mM NaCl, 20 mM imidazole, pH 7.5; hMAT2A: 50 mM Tris–HCl pH 8, 50 mM NaCl, 10% glycerol) and subsequently lysed by sonication, then clarified by centrifugation.
Soluble cell lysates were purified by Ni-NTA column chromatography. The Ni-NTA column was first pre-equilibrated in lysis buffer, prior to loading the soluble lysate. The desired protein was then eluted in a series of washes with lysis buffer of increasing imidazole concentration: 8 column volumes (CV) of lysis buffer containing 30 mM imidazole, 8 CV of lysis buffer containing 60 mM imidazole then 5 CV lysis buffer containing 250 mM imidazole to elute the protein. All fractions were analysed by SDS-PAGE. The purified protein fractions which were eluted with 250 mM imidazole were then exchanged into appropriate buffers for storage (CmoA: 20 mM HEPES, 150 mM KCl, pH 7.5; COMT: 50 mM potassium phosphate buffer, 300 mM NaCl, 10% glycerol v/v, pH 7.4; CNMT: 100 mM potassium phosphate buffer, 200 mM NaCl, 10% glycerol v/v, pH 7; CM: 10 mM potassium phosphate buffer, 160 mM NaCl, pH 7.5; hMAT2A: 50 mM Tris–HCl pH 8, 50 mM NaCl, 10% glycerol) and concentrated by centrifugation (7000 rpm) using Vivaspin 20 centricon, of either MWCO 10,000 or 30,000 (Sartorius Stedim Biotech). Purified enzymes were either used immediately or stored at -80 ˚C.

Site-directed mutagenesis CNMT & COMT. The pET28a-CNMT[5] construct was used as a template for site-directed mutagenesis and codon changes were introduced at either Y81, E204 or E207 amino acid positions using mutagenic primers (see **Table S1**) based on a model of cxSAM within the CNMT active site (model produced through overlaying cxSAM over the coordinates of SAH within the crystal structure PDB 6GKV using PyMOL 1.3 software). Mutagenesis was conducted using standard PCR techniques, after which *DpnI* restriction endonuclease was added to ensure the digestion of parental wild-type plasmids. Mutant plasmids were then used to transform *E.coli* DH5α chemically competent cells. Following amplification, plasmids were extracted using the QIAprep Spin MiniPrep protocol, and verified by nucleotide sequencing (GATC Biotech).
The pET21-COMT [23] construct was used as a template for site-directed mutagenesis and codon changes were introduced at the M40 amino acid position using mutagenic primers (see **Table S1**). Mutagenesis was conducted using standard NEB Q5 mutagenesis protocols, after which *KLD* restriction endonuclease was added to ensure the digestion of parental wild-type DNA. Digested DNA was subsequently was used to transform *E.coli* DH5α chemically competent cells for DNA amplification, extracted and verified as above.

Chorismate production using *E. coli* KA12: Growth, accumulation conditions and purification. The CM-deficient *E.coli* KA12 strain was kindly provided by Prof. Dr. Donald Hilvert (ETH Zurich) for chorismate production, which was carried out using the methods previously developed by Grisostomi *et al.*[28]

Crude chorismate was purified using **HPLC method 7** which uses a Shimadzu semi-preparative HPLC; a Phenomenex Gemini C18 column (bead size of 5 μM, pore size of 110Ä, 250 x 10 mm, flow rate 5 mL min^{-1}); with a gradient of 0-5 min 3% B, 5-30 min 3-20% B, 30-30.3 min 20-95% B, 30.3-33 min 95% B, 33-33.5 min 95-5% B, 33.5-38 min 5% B. Mobile phase A: water + 0.05% TFA; mobile phase B: acetonitrile + 0.05% TFA. HPLC fractions were combined, lypholised and stored at -80 ˚C until use. The chorismate sample was subsequently analysed by LC-MS and NMR.

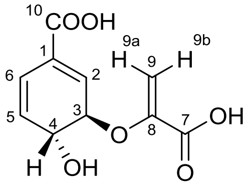

^{1}H NMR (400 MHz, MeOD). δ 6.87 (m, 1H, *H2*), δ 6.35 (m, 1H, *H6*), δ 6.0 (dd, *J* = 10.0, 2.8, 1H, *H5*), δ 5.50 (d, *J* = 3.0, 1H, *H9a*), δ 4.83 (d, *J* = 3.0, 1H, *H9b*), δ 4.71 (m, 1H, *H4*). ^{13}C NMR (101 MHz, MeOD) δ 167.5 (*C10*), 166.5 (*C7*), 151.0 (*C8*), 134.5 (*C2*), 133.4 (*C5*), 130.8 (*C1*), 122.7 (*C6*), 97.1 (*C9*), 81.8 (*C3*), 71.2 (*C4*). HRMS ESI +ve, calculated mass for $C_{10}H_{10}NaO_6^+$ $[M+Na]^+$: 249.0370; mass found: 249.0367.

Chorismate mutase activity assays. Chorismate mutase activity was initially monitored by following the disappearance of the UV absorbance of chorismate at 275 nm. The UV spectra was first measured for standards of prephenate, chorismate and phenylpyruvate, and the 275 nm wavelength identified as suitable to monitor the conversion of chorismate (maximum absorbance at 275 nm) to prephenate (no visible UV spectrum). Assays were carried out using chorismate (1 mM) and chorismate mutase (0.5-10 μM) in potassium phosphate buffer (10 mM, 160 mM NaCl, pH 7.5). Assays were incubated at 30 ˚C for 30 minutes, with readings of UV absorbance (275 nm) taken every minute. Control assays with either ‘no chorismate’ or ‘no chorismate mutase’ were also recorded, with the ‘no chorismate’ control representing the spontaneous rearrangement of chorismate to prephenate.

Assay Methods

COMT WT and Y200L assays with synthetic cxSAM (Figure 2a). Analytical assays were carried out in 100 μL volume with synthetic cxSAM (1.6 mM), substrate **2** or **3** (0.25 mM), COMT (100 μM), $MgCl_2$ (6 mM), DTT (1 mM) in potassium phosphate buffer (50 mM, pH 7.4). Assays were incubated at 37 ˚C with constant agitation (650 rpm) for 20 h, and subsequently quenched with an equal volume of methanol and clarified by centrifugation prior to analysis by **HPLC method 1** which uses a Shimadzu analytical UHPLC (model: UFLC-XR); a Phenomenex Kinetex C18 column (bead size of 5 μM, pore size of 100Å, 100 x 4.6 mm, flow rate 1.5 mL min^{-1}); with a gradient of 0-1 min 5-20% B, 1-5 min 20-23% B, 5-5.5 min 23-95% B, 5.5-6 min 95% B, 6-6.1 min 95-5% B, 6.1-7 min 5% B. Mobile phase A: water + 0.05% TFA; mobile phase B: acetonitrile + 0.05% TFA. Assay peaks were assigned by comparison with the authentic synthetic or commercial standards. Products were also confirmed by LC-MS analysis (Waters AQUITY LC and Orbitrap LTQ MS).

An assay of COMT with **2** and cxSAM was conducted on a 10 mL scale for product characterisation, with synthetic cxSAM (3 mM), **2** (2 mM), $MgCl_2$ (6 mM), COMT Y200L mutant (200 μM), DTT (1 mM) in potassium phosphate buffer (50 mM, pH 7.4). The assay was incubated at 37 ˚C with constant agitation (650 rpm) for 20 h and the carboxymethylated product was purified by **HPLC method 2** which uses a Shimadzu semi-preparative HPLC; a Phenomenex Gemini C18 column (bead size of 5 μM, pore size of 110Ä, 250 x 10 mm, flow rate 5 mL min^{-1}); with a gradient of 0-6 min 5-20% B, 6-17 min 20% B, 17-18 min 20-95% B, 18-23 min 95% B, 23-24 min 95-5% B, 24-30 min 5% B. Mobile phase A: water + 0.05% TFA; mobile phase B: acetonitrile + 0.05% TFA. Fractions corresponding to the carboxymethylated product were then lyophilized, yielding 1.0 mg of purified product (26% isolated yield). The purified product was then characterized by mass spectrometry (negative electrospray), NMR (^{1}H, 2D COSY) and UV analysis.

WT CNMT assays with synthetic cxSAM (Figure 2b). For assays with THIQ **4**, **6** and **7**, the CNMT enzyme was pre-incubated with THIQ **5** (0.2 mM) in reaction buffer (potassium phosphate buffer (100 mM, pH 7)) at 30 ˚C with constant agitation (800 rpm) for 1 h to deplete any co-purified SAM. The enzyme was then concentrated using a 30,000 MWCO centricon, and washed three times with reaction buffer. Conversion assays were then conducted with synthetic cxSAM (1.6 mM), substrate **4**, **6** and **7** (0.25 mM), CNMT (100 μM) in potassium phosphate buffer (100 mM, pH 7). Assays were initiated by the addition of substrate, incubated at 30 ˚C (800 rpm) for 20 h and subsequently quenched as above. Assays with THIQ **5**, were conducted in an identical manner, except the enzyme was initially pre-incubated with **4**. All assays were then analysed by **HPLC method 3** which uses a Shimadzu analytical UHPLC (model: UFLC-XR); a Phenomenex Kinetex C18 column (bead size of 5 μM, pore size of 100 Å, 250 x 4.6 mm column, flow rate: 1 mL min^{-1}); with a gradient of 0-12 min 17% B. Mobile phase A: water + 0.05% TFA; mobile phase B: acetonitrile + 0.05% TFA. For assays conducted with substrates **6** and **7**, the following gradient was used: 0-1 min 5% B, 1-2 min 5-10% B, 2-8 min 10-33% B, 8-8.1 min 33-95% B, 8.1-9 min 95% B, 9-9.5 min 95-5% B, 9.5-13 min 5% B.

CNMT mutant assays (Figure S4). WT CNMT and CNMT mutants were assayed for activity with **4** and either SAM or cxSAM. The assays were carried out using SAM (1 mM) or synthetic cxSAM (1.6 mM), **4** (0.25 mM), CNMT enzyme (100 μM) in potassium

phosphate buffer (100 mM, pH 7), in a total volume of 100 µl. Assays were incubated at 30 °C, with constant agitation (800 rpm) and were subsequently quenched as above and analysed by **HPLC method 4** which uses a Shimadzu analytical UHPLC (model: UFLC-XR); a Phenomenex Kinetex C18 column (bead size of 5 µM, pore size of 100 Å, 250 x 4.6 mm column, flow rate: 1 mL min^{-1}); with a gradient of 0-13.5 min 7% B. Mobile phase A: water + 0.05% TFA; mobile phase B: acetonitrile + 0.05% TFA.

Time course assays with CNMT, THIQ 4 and SAM or cxSAM (Figure 3b). Assays comprising SAM (1 mM) or synthetic cxSAM (1.6 mM), THIQ **4** (0.25 mM), CNMT WT, Y81R or Y81K (100 µM) in potassium phosphate buffer (100 mM, pH 7), in a total volume of 100 µl, were incubated at 37 ˚C (800 rpm). Reactions were quenched at t = 1, 30, 60, 120, 240 min as above and analysed by **HPLC method 4**.

Competition assays with CNMT enzymes and varying ratios of SAM:cxSAM (Figure S6). Assays were conducted at varying ratios of SAM:cxSAM (1: 2, 1: 4, 1: 8 & 1: 9 mM). Given that commercial SAM is enantiomerically pure (*S*-configured sulphonium epimer) and synthetic cxSAM is a mixture of sulphonium epimers, this equates to the reported ratios of 1:1, 1:2, 1:4, 1:4.5. Assays were carried out using THIQ **4** (0.5 mM), either WT, Y81R or Y81K CNMT in potassium phosphate buffer (100 mM, 160 mM NaCl, pH 7). Assays were conducted at 30 ˚C, with constant agitation (800 rpm) and quenched after 5 h as above and analysed by **HPLC method 3**.

COMT mutant assays (Figure 3c). COMT mutant activity with cxSAM was assessed for the substrate **2**. The COMT enzyme (0.2 mM) was pre-incubated with **3** (0.2 mM,) $MgCl_2$ (6 mM), DTT (1 mM) in potassium phosphate buffer (50 mM, pH 7.4) at 37 ˚C with constant agitation (800 rpm) for 1 h. Following isolation using an Amicon Ultra-15 (30,000 MW), assays were carried out using pre-incubated COMT (100 µM), SAM (1 mM) or synthetic cxSAM (1.6 mM), **2** (0.25 mM), $MgCl_2$ (6 mM), DTT (1 mM) in potassium phosphate buffer (50 mM, pH 7.4). Assays were incubated at 37 ˚C with constant agitation (800 rpm) for 5 or 17 h, and were subsequently quenched as above and analysed using **HPLC method 1**.

Optimisation of CmoA Catalysed cxSAM Formation (Figure S9): The optimisation assays for the conversion of SAM to cxSAM, were carried out using chorismate (2-6 mM), CM (0-1 µM), SAM (0.5 mM), and CmoA (100-200 µM) in potassium phosphate buffer (100 mM, pH 8). Assays were conducted at 37 ˚C with constant agitation (800 rpm) and quenched after 5 h as above and analysed using **HPLC method 5** which uses a Shimadzu analytical UHPLC (model: UFLC-XR); a Phenomenex Luna HILIC column (bead size of 5 µM, pore size of 200 Å, 150 x 4.6 mm, flow rate 1.5 mL/min) with a gradient of 0-2.5 min 90% B, 2.5-10 min 90-50% B, 10-12.5 min 50-10% B, 12.5-17 min, 10% B. Mobile phase A: ammonium formate buffer (pH 3.3); mobile phase B: acetonitrile + 0.05% TFA. Enzymatic cxSAM was confirmed by comparison to the synthetic standard and co-injection with the synthetic standard.

Optimisation of CmoA and CNMT tandem assay (Figure 4b). Optimisation assays for the tandem assay involving CmoA and CNMT WT or Y81R mutant were carried out in two parts; part 1: SAM (0.5 mM), chorismate (4-6 mM) and CmoA (100-400 µM) in potassium phosphate buffer (100 mM, pH 8) were incubated at 37 ˚C with constant agitation (800 rpm) for 5 h. Part 2: **4** (0.1 – 0.25 mM) and WT or Y81R CNMT (100 µM) were added and assays were further incubated at 37 ˚C with constant agitation (800 rpm) for 17 h. Assays were subsequently quenched as above and analysed using **HPLC method 4**.

CNMT activity assay with cxSAE (Figure 5). CNMT activity was assessed with THIQ **4** and synthetic cxSAE, using CNMT that had first been preincubated with **5**, as described previously. The assays were carried out using CNMT (100 µM), synthetic cxSAE (1.6 mM) and **4** (0.25 mM) in potassium phosphate buffer (100 mM, pH 7). Assays were incubated at 37 ˚C with constant agitation (800 rpm) for 18 h, and were subsequently quenched as above and analysed using **HPLC method 3**. For chiral analysis, assays were analysed using **HPLC method 6** which uses a Shimadzu analytical UHPLC (model: UFLC-XR); a Phenomenex Lux i-Cellulose-5 column (bead size of 5 µM, 4.6 x 250 mm column, flow rate: 1 mL min^{-1}) with a gradient of 0-12 min 80% B. Mobile phase A: water + 0.1% FA; mobile phase B: acetonitrile + 0.1%FA.

Tandem coupled assay with hMAT and ComA to generate cxSAE (Figure 5). Assays were carried out using ATP (0.5 mM), DTT (1 mM), L-ethionine (0.5 mM), hMAT2A I322V (68 µM) and $MgCl_2$ (5 mM) in potassium phosphate buffer (100 mM, pH 7). The reaction mixture was incubated at 37 ˚C with constant agitation (800 rpm) for 1 h, after which point prephenate (1.25 mM) and CmoA (50 µM) were added. The assays were further incubated at 37 ˚C with constant agitation (800 rpm) for 180 mins, and were subsequently quenched as above and analysed using **HPLC method 5**.

Coupled assay with hMAT, CmoA and CNMT (Figure S15) Assays were carried out using ATP (0.5 mM), DTT (1 mM), L-methionine or L-ethionine (0.5 mM), hMAT2A I322V (68 µM) and $MgCl_2$ (5 mM) in potassium phosphate buffer (100 mM, pH 7). The reaction mixture was incubated at 37 ˚C with constant agitation (800 rpm) for 1 h, after which point prephenate (1.25 mM) and CmoA (50 µM) were added. The assays were further incubated for 4 h. To circumvent protein precipitation, the assay mixtures were passed through a Vivaspin 20 centrifugal concentrator, of MWCO 30,000 (Sartorius Stedim Biotech) to remove protein prior to the addition of **4** (0.1 mM) and CNMT Y81R (100 µM, pre-incubated with **5** as above). The assays were incubated at 30 ˚C with constant agitation (800 rpm) for 17 h. Assays were subsequently quenched as above and analysed using **HPLC method 5** and compared to synthetic standards.

Synthesis of cxSAM

Synthetic procedure 1: *S*-Alkylation of SAH. The chemical synthesis of SAM analogues was based on a procedure reported by Kim *et al*.[21] SAH (10 mg, 0.04 mM) was dissolved in 2.5 mL of 150 mM ammonium bicarbonate solution, prior to the addition of 500 mg of the bromo-carboxylic acid. The mixture was then incubated with constant agitation (37 ˚C, 18 h) and subsequently purified using **HPLC method 8** which uses a Shimadzu semi-preparative HPLC; a Phenomenex Luna HILIC column (bead size of 5 μM, pore size of 200 Å, 150 x 21.2 mm, flow rate 10 mL min^{-1}); with a gradient of 0-10 min 10% B, 10-13 min 10-50% B, 13-13.1 min 50-10% B. Mobile phase A: 5 mM ammonium formate buffer (pH 3.3); mobile phase B: acetonitrile + 0.05% TFA.

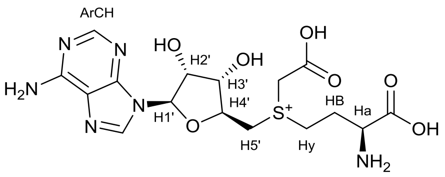

1 (88%)

Carboxy-*S*-adenosyl-L-methionine (1) was prepared according to **synthetic procedure 1** using 2-bromoacetic acid. HPLC fractions were lyophilised to yield **1** as a white solid (10.18 mg, 23.0 μmol, 88 %). ^{1}H NMR (400 MHz, D_2O) δ 8.40 (s, 1H), 8.37 (s, 1H), 6.12 (m, 1H), 4.86 (m, 1H), 4.56 (m, 1H), 3.98 (m, 2H), 3.80 (m, 1H), 3.58 (m, 2H), 2.30 (m, 2H); HRMS ESI +ve, calculated mass for $C_{16}H_{23}N_6O_7S^+$ $[M]^+$: 443.1343; mass found: 443.1349.
The concentration of CxSAM was determined by measuring UV-Absorbance at 260 nm (NanoDrop2000 Spectrophotometer, ThemoScientific), and assuming an extinction coefficient equivalent to that of SAM (ε260 = 15.4 $cm^{-1}mM^{-1}$). A standard curve of UV absorbance versus SAM concentration was used to determine the concentration of CxSAM.

Synthesis of Carboxymethylated-Catechol Synthetic Standards

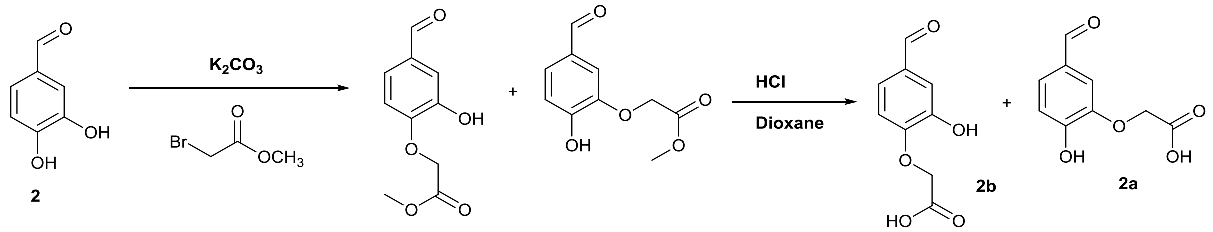

2-(4-formyl-2-hydroxyphenoxy)acetic acid (2a) & 1-(4-formyl-2-hydroxyphenoxy)acetic acid (2b) were prepared through the addition of methyl-2-bromoacetate (3.52 g, 23 mmol, 1.0 eq) to a suspension of **2** (3.14 g, 23 mmol, 1.0 eq) and K_2CO_3 (3.14 g, 23 mmol, 1.0 eq) in acetone (100 mL). The mixture was heated to reflux (60 ˚C) for 5 h, after which the reaction was filtered and then concentrated *in vacuo*. The resultant residue was re-dissolved in diethyl ether (50 mL) and water (50 mL). The mixture was acidified to pH 3 (using 50% aqueous sulphuric acid) and product extracted into diethyl ether (2 x 50 mL). The organic fractions were combined, dried over $MgSO_4$ and concentrated *in vacuo* to yield the crude methyl ester.
The crude methyl ester (150 mg) was added to a mixture of 10 mL dioxane and 3 mL 12 M HCl. The mixture was heated at reflux for 6 h, after which the solvent was removed *in vacuo* to afford the crude product (**2a/2b**) as a brown/ yellow solid. The residue was dissolved in 1 mL water and the *meta*- and *para*- products were isolated using **HPLC method 9** which uses a Shimadzu semi-preparative HPLC (LC-20AP); a Phenomenex Gemini C18 column (bead size of 5 μM, pore size of 110Ä, 250 x 10 mm, flow rate 5 mL min^{-1}); with a gradient of 0-4 min 5% B, 4-5 min 5-15% B, 5-20 min 15-35% B, 20-22 min 35-95%, 22-26 min 95%, 26-28 min 95-5% B, 28-32 min 5% B. Mobile phase A: 5 mM ammonium bicarbonate buffer (pH 7.2); mobile phase B: acetonitrile. If required, further purification was achieved using the following method: 0-4 min 5% B, 4-5 min 5-20% B, 5-20 min 20-23% B, 20-22 min 23-95% B, 22-26 min 95% B, 26-28 min 95-5% B, 28-32 min 5% B. Mobile phase A: 95% water with 0.05% TFA; mobile phase B: acetonitrile.

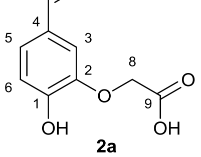

2a

^{1}H (400 MHz, D_2O) δ 9.67 (s, 1H, H7), 7.55 (d, *J* = 8.2 Hz, 1H, H5), 7.41 (s, 1H, H3), 7.06 (d, *J* = 8.2 Hz, 1H, H6), 4.64 (s, 2H, H8). HRMS ESI -ve, calculated mass for $C_9H_8O_5^-$ $[M-H]^-$: 195.0299; mass found: 195.0294.

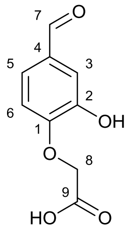

2b (32%)

(45 mg, 32%); ^{1}H NMR (400 MHz, D_2O) δ 9.69 (s, 1H, *H7*), 7.50 (dd, *J* 8.4, 1H, 2.0, *H5*), 7.40 (d, *J* 2.0, 1H, *H3*), 7.04 (d, *J* 8.4, 1H, *H6*), 4.78 (s, 2H, *H8*). ^{13}C NMR (126 MHz, D_2O) δ 195.0 (*C7*), 174.1 (*C9*), 151.9 (*C2*), 145.6 (*C1*), 130.1 (*C4*), 126.1 (*C5*), 115.0 (*C3*), 112.6 (*C6*), 66.2 (*C8*). HRMS ESI +ve, calculated mass for $C_9H_9O_5^+$ [M+H]$^+$: 197.0444; mass found: 197.0442.

Synthesis of Carboxymethylated-Tetrahydroisoquinoline Synthetic Standards

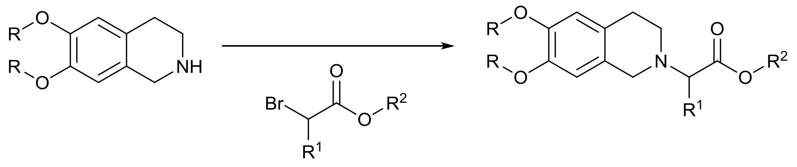

Synthetic procedure 2: *N*-Alkylation of tetrahydroisoquinolines using Br-R. Potassium carbonate (8.68 mmol, 2.0 eq) and acetonitrile (13 mL) were added to a flask containing the THIQ (4.38 mmol, 1.0 eq). Br-R (1.5 eq) was then added and the reaction heated at reflux overnight. Solvent was then removed *in vacuo* and the resultant residue dissolved in ethyl acetate (100 mL). After washing with water (100 mL) and brine (100 mL), the organic phase was dried over $MgSO_4$ and concentrated *in vacuo* to afford a yellow oil.

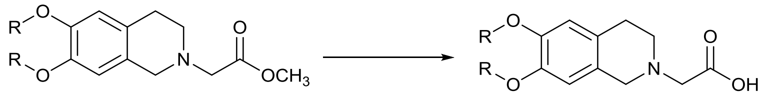

Synthetic procedure 3: Hydrolysis of *N*-acetate tetrahydroisoquinoline. The ester product from **synthetic procedure 2** (90 mg) was added to dioxane (10 mL) and 12 M HCl (3 mL). After heating at reflux for 6 h, the reaction was cooled and solvent removed *in vacuo*. The resultant residue was then dissolved in water (30 mL) prior to loading onto an Agilent C18 BondElut which had been activated with methanol (3 column volumes (CV)) and equilibrated with water (3 CV). After washing with water (3 CV), organics were eluted with methanol (3 CV) and then concentrated *in vacuo*.

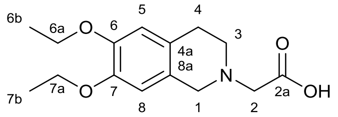

5a (47%)

***N*-acetic acid-(6,7-diethoxy)-1,2,3,4-tetrahydroisoquinoline (5a)** was prepared from **5** and methyl-2-bromoacetate according to synthetic procedures 2 and 3. Purification was carried out using **HPLC method 10** which uses a Shimadzu semi-preparative HPLC (LC-20AP); a Phenomenex Gemini C18 column (bead size of 5 μM, pore size of 110Ä, 250 x 10 mm, flow rate 5 mL min^{-1}); with a gradient of 0-4 min 5% B, 4-5 min 5-16% B, 5-20 min 16% B, 20-22 min 16-95% B, 22-26 min 95% B, 26-27 min 95-5% B, 26-30 min 5% B. Mobile phase A: water + 0.05% TFA; mobile phase B: acetonitrile + 0.05% TFA. Purification yielded the title compound **5a** as an off-white solid (40 mg, 47%). ^{1}H NMR (400 MHz, MeOD) δ 6.68, 6.60 (2 x s, 2H, *H8*, *H5*), 4.28 (s, 2H, *H1*), 4.05 (d, *J* = 2.8, 2H, *H2*), 3.93 – 3.86 (m, 4H, *H6a, H7a*), 3.61 - 3.36 (m, 2H, *H3*), 3.00 (d, *J* 7.6, 2H, *H4*), 1.28 – 1.21 (m, 6H, *H6b*, *H7b*). ^{13}C NMR (101 MHz, $CDCl_3$) δ 167.7 (*C3a*), 149.0, 148.3 (*C6, C7*), 122.0, 117.9 (*C4a, C8a*), 112.9, 111.3 (*C5, C8*) 64.7, 64.6 (*C6a, C7a*), 54.1 (*C1*), 53.0 (*C2*), 50.2 (*C3*), 23.6 (*C4*), 14.7, 14.7 (*C6b, C7b*). HRMS ESI +ve, calculated mass for $C_{15}H_{22}NO_4^+$ [M+H]$^+$: 280.1543; mass found: 280.1530.

Synthesis of cxSAE

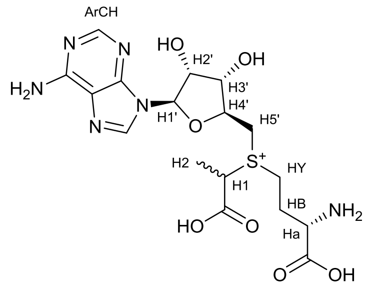

8 (42%)

(±)-Carboxy-*S*-adenosyl-ethionine (8) was prepared according to **synthetic procedure 1** using (±)-2-bromopropionic acid. HPLC fractions were lypholised to yield **8** as a white solid (5.0 mg, 10.93 µmol, 42%). ^{1}H NMR (400 MHz, D_2O) δ 8.42 (1 H, s, ArCH), 8.23 (1 H, m, ArCH), 6.09 (1 H, m, H1'), 4.94 (1 H, m, H2'), 4.54 (1 H, m, H3'), 4.39 (1 H, m, H4'), 3.92 (2 H, m, H5'), 3.68 (1 H, m, H*α*), 3.41 (2 H, m, H*γ*), 2.25 (2 H, m, Hβ), 1.62 (3 H, m, H2); HRMS ESI +ve, calculated mass for $C_{17}H_{25}N_6O_7S^+$ $[M]^+$: 457.1505; mass found: 457.1520.

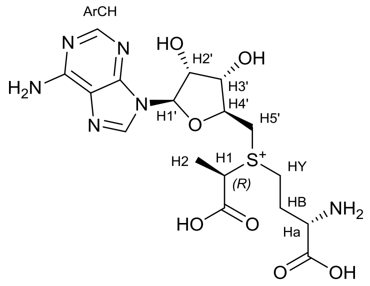

9 (36%)

(*R*)-Carboxy-*S*-adenosyl-ethionine (9) was prepared according to **synthetic procedure 1** using (*S*)-2-bromopropionic acid. HPLC fractions were lyophilised to yield **9** as a white solid (4.3 mg, 9.40 µmol, 36%). ^{1}H (400 MHz, D_2O) δ 8.45 (2 H, m, ArCH & ArCH), 6.18 (1 H, m, H1'), 4.93 (1 H, m, H2'), 4.62 (1 H, m, H3' & H1), 4.44 (1 H, m, H4'), 3.92 (2 H, m, H5'), 3.77 (1 H, m, Hα), 3.52 (2 H, m, Hγ), 2.32 (2 H, m, Hβ), 1.57 (3 H, m, H2); HRMS ESI +ve, calculated mass for $C_{17}H_{25}N_6O_7S^+$ $[M]^+$: 457.1505; mass found: 457.1502.

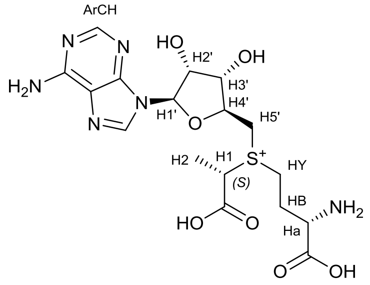

10 (50%)

(*S*)-Carboxy-*S*-adenosyl-ethionine (10) was prepared according to **synthetic procedure 1** using (*R*)-2-bromopropionic acid. HPLC fractions were lyophilised to yield **10** as a white solid (5.9 mg, 12.9 µmol, 50%). ^{1}H (400 MHz, D_2O) δ 8.44 (2 H, m, ArCH), 6.18 (1 H, m, H1'), 4.92 (1 H, m, H2'), 4.62 (1 H, m, H3' & H1), 4.43 (1 H, m, H4'), 3.92 (2 H, m, H5'), 3.76 (1 H, m, Hα), 3.53 (2 H, m, Hγ), 2.31 (2 H, m, Hβ), 1.66 (3 H, m, H2); HRMS ESI +ve, calculated mass for $C_{17}H_{25}N_6O_7S^+$ $[M]^+$: 457.1505; mass found: 457.1506.

Synthesis of Carboxyethylated-Tetrahydroisoquinoline Synthetic Standards

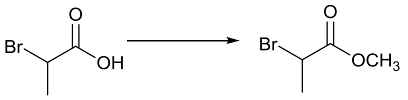

Synthetic procedure 4: Methylation of 2-Bromopropionic acid. 2-Bromopropionic acid (1.9 eq) was dissolved in methanol (5 mL). Trimethylsiliyl diazomethane (2 M in hexane) was added dropwise with stirring until the yellow colour persisted. The reaction was concentrated *in vacuo* to an off white oil. Potassium carbonate (2.0 eq) and acetonitrile (6.5 mL) were added to the crude oil. 6,7-

dimethoxy-1,2,3,4-tetrahydroisoquinoline (1.0 eq) was then added and the reaction heated at reflux overnight. Solvent was then removed *in vacuo* and the resultant residue dissolved in ethyl acetate (50 mL). After washing with water (50 mL) and brine (50 mL), the organic phase was dried over $MgSO_4$ and concentrated *in vacuo* to afford 2-Bromopropionate as a yellow oil.

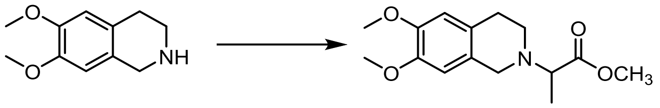

Synthetic procedure 5: *N*-alkylation of tetrahydroisoquinolines. The THIQ was dissolved in 6.5 mL of acetonitrile, prior to the addition of the 2-Bromopropionate product from **synthetic procedure 4** and K_2CO_3 (2 eq). The mixture was refluxed for 18 hours before being cooled to room temperature and concentrated *in vacuo*. The resultant residue was dissolved in water (20 mL) and washed with ethyl acetate (3 x 15 mL). The organic fractions were combined and purified on via silica column chromatograhy (10% methanol in DCM).

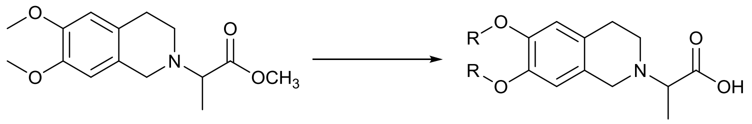

Synthetic procedure 6: Hydrolysis of *N*-2-propionate tetrahydroisoquinolines. The chirality of the product from **synthetic procedure 5** was tested using chiral **HPLC method 11** which uses a Shimadzu analytical HPLC; a Diacel chiralcel OD-R column (bead size of 5 μM, 4.6 x 250 mm, flow rate of 1 ml min^{-1}); with an isocratic gradient of 0-20 min 75% B. Mobile phase A: water; mobile phase B: methanol. Once enantiopurity was confirmed, the oil was dissolved in 5.5 mL water:THF (1:2) and LiOH (3.7 eq) and left to stir for 4 hours. THF was removed *in vacuo* and the resultant aqueous solution acidified to pH 2 with 1 M HCl and washed with DCM (3 x 5 mL). The organic fraction was dried over Na_2SO_4 and concentrated *in vacuo* to yield an off white solid.

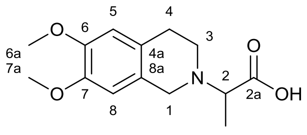

4c (44%)

***N*-2-propionic acid-(6,7-dimethoxy)-1,2,3,4-tetrahydroisoquinoline (4c)** was prepared from *(±)*-2-bromopropionic acid (1.8 g, 12.9 mmol) and **4** (1.2 g, 4.3 mmol) according to **synthetic procedures 4, 5 and 6** and yielded **4c** as an off-white solid (720 mg, 44%). ^{1}H NMR (400 MHz, MeOD) δ 6.63, 6.59 (2 x s, 2H, H5, H8), 3.81-3.58 (m, 8H, H1, H6a, H7a), 3.05 (q, *J* = 6.9 Hz, 1H, H2), 2.96-2.67 (m, 4H, H3, H4), 1.32 (d, *J* = 6.9 Hz, 3H, H2b); ^{13}C NMR (101 MHz, MeOD) 181.0 (C2a), 148.8, 148.5 (C6, C7) 128.4, 127.8 (C4a, C8a), 112.9, 111.2 (C5, C8), 67.5 (C2), 56.4, 56.4 (C6a, C7a), 53.5 (C1), 29.5 (C4), 16.6 (C2b). HRMS ESI +ve, calculated mass for $C_{14}H_{20}NO_4^+$ $[M+H]^+$: 266.1387; mass found: 266.1374.

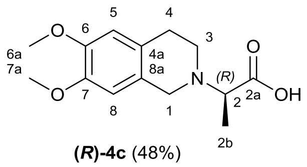

(*R*)-4c (48%)

***(R)*-*N*-2-propionic acid-(6,7-dimethoxy)-1,2,3,4-tetrahydroisoquinoline ((*R*)-4c)** was prepared from *(S)*-2-bromopropionic acid (1.4 g, 9.8 mmol)and **4** (634 mg, 3.3 mmol) according to **synthetic procedures 4, 5 and 6** and yielded **(*R*)-4c** in 95.1 % ee as an off-white solid (416 mg, 48%). ^{1}H NMR (400 MHz, MeOD) δ 6.69, 6.65 (2 x s, 2H, H5, H8), 4.12 (m, 2H, H1), 3.76 – 3.67 (d, 6H, H6a, H7a), 3.48 (q, *J* = 7.0 Hz, 1H, H2), 3.26 (m, 2H, H3), 2.94 (q, *J* = 6.1 Hz, 2H, H4), 1.45 (d, *J* = 7.0 Hz, 3H, H2b); ^{13}C NMR (101 MHz, MeOD) 180.3 (C2a), 150.0, 149.5 (C6, C7) 125.6, 123.2 (C4a, C8a), 112.6, 111.0 (C5, C8), 66.6 (C2), 56.5, 56.5 (C6a, C7a), 52.3 (C1), 27.3 (C4), 14.6 (C2b); HRMS ESI +ve, calculated mass for $C_{14}H_{20}NO_4^+$ $[M+H]^+$: 266.1387; mass found: 266.1378.

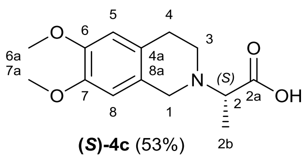

(*S*)-4c (53%)

***(S)*-*N*-2-propionic acid-(6,7-dimethoxy)-1,2,3,4-tetrahydroisoquinoline ((*S*)-4c)** was prepared from *(R)*-2-bromopropionic acid (0.3 g, 2.1 mmol) and **4** (0.13 g, 0.7 mmol) according to **synthetic procedures 4, 5 and 6** and yielded **(*S*)-4c** 94.6 % ee as an off-white solid (92 mg, 53%). ^{1}H NMR (400 MHz, MeOD) δ 6.66, 6.62 (2 x s, 2H, H5, H8), 3.83-3.59 (m, 8H, H1, H6a, H7a), 3.07 (q, *J* = 6.8 Hz,

1H, H2), 2.98-2.70 (m, 4H, H3, H4), 1.34 (d, *J* = 6.9, 3H, H2b); ^{13}C NMR (101 MHz, MeOD) 181.0 (C2a), 148.8, 148.6 (C6, C7) 128.2, 127.8 (C4a, C8a), 112.8, 111.1 (C5, C8), 67.5 (C2), 56.5, 56.4 (C6a, C7a), 53.5 (C1), 29.4 (C4), 16.6 (C2b); HRMS ESI +ve, calculated mass for $C_{14}H_{20}NO_4^+$ $[M+H]^+$: 266.1387; mass found: 266.1380.

Supplementary Figures

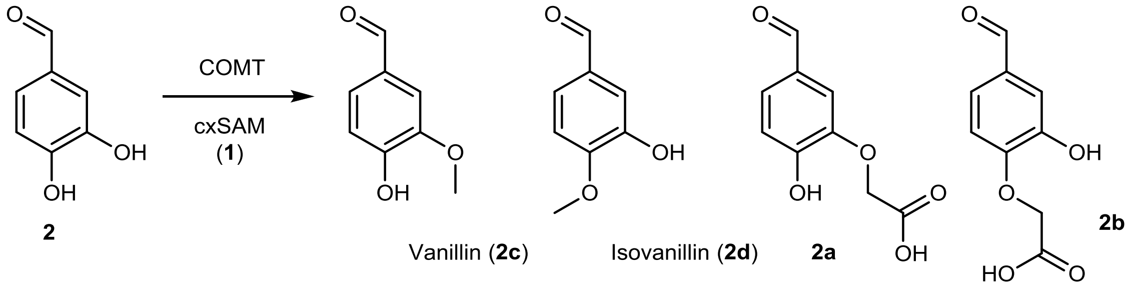

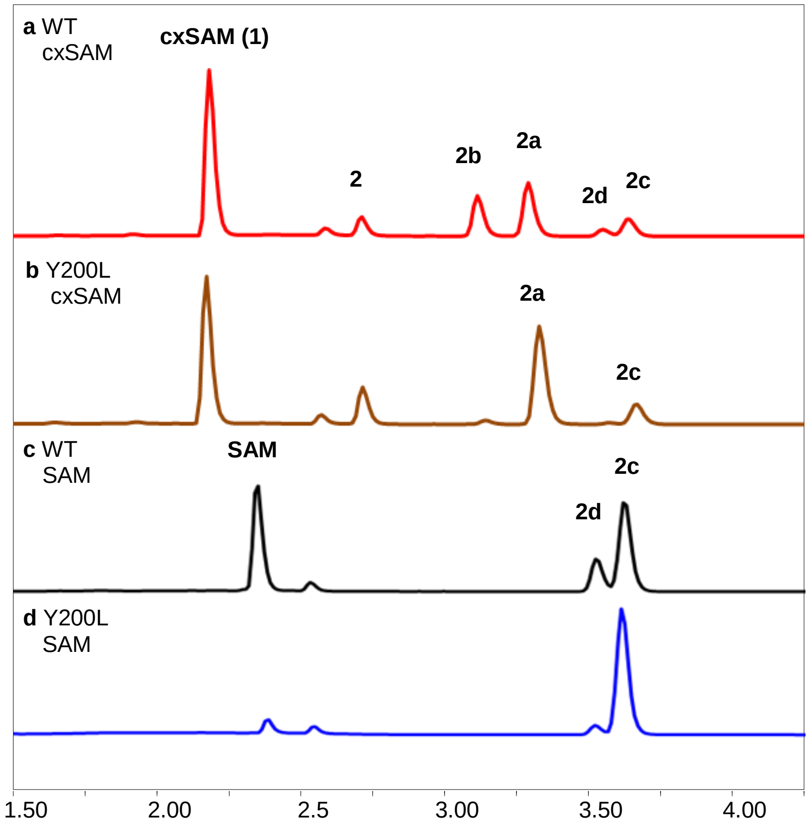

Figure S1. C18 RP HPLC analysis of enzyme activity assays of substrate DHBAL (**2**) with: (**a**) WT COMT and cxSAM (red); (**b**) COMT Y200L and cxSAM (brown); (**c**) WT COMT with SAM (black); and (**d**) COMT Y200L and SAM (blue). The assays were conducted using synthetic cxSAM (1.6 mM) or SAM (Sigma)(1 mM), substrate **2** (0.25 mM), COMT (100 µM), $MgCl_2$ (6 mM), DTT (1 mM) in potassium phosphate buffer (50 mM, pH 7.4), incubated at 37 ˚C for 20 hours. 15 ±1% **2c** and 6 ±1% **2d** methylation was observed within WT and cxSAM assays and 15 ±1% **2c** and 1 ±1% **2d** methylation was observed within Y200L and cxSAM assays.

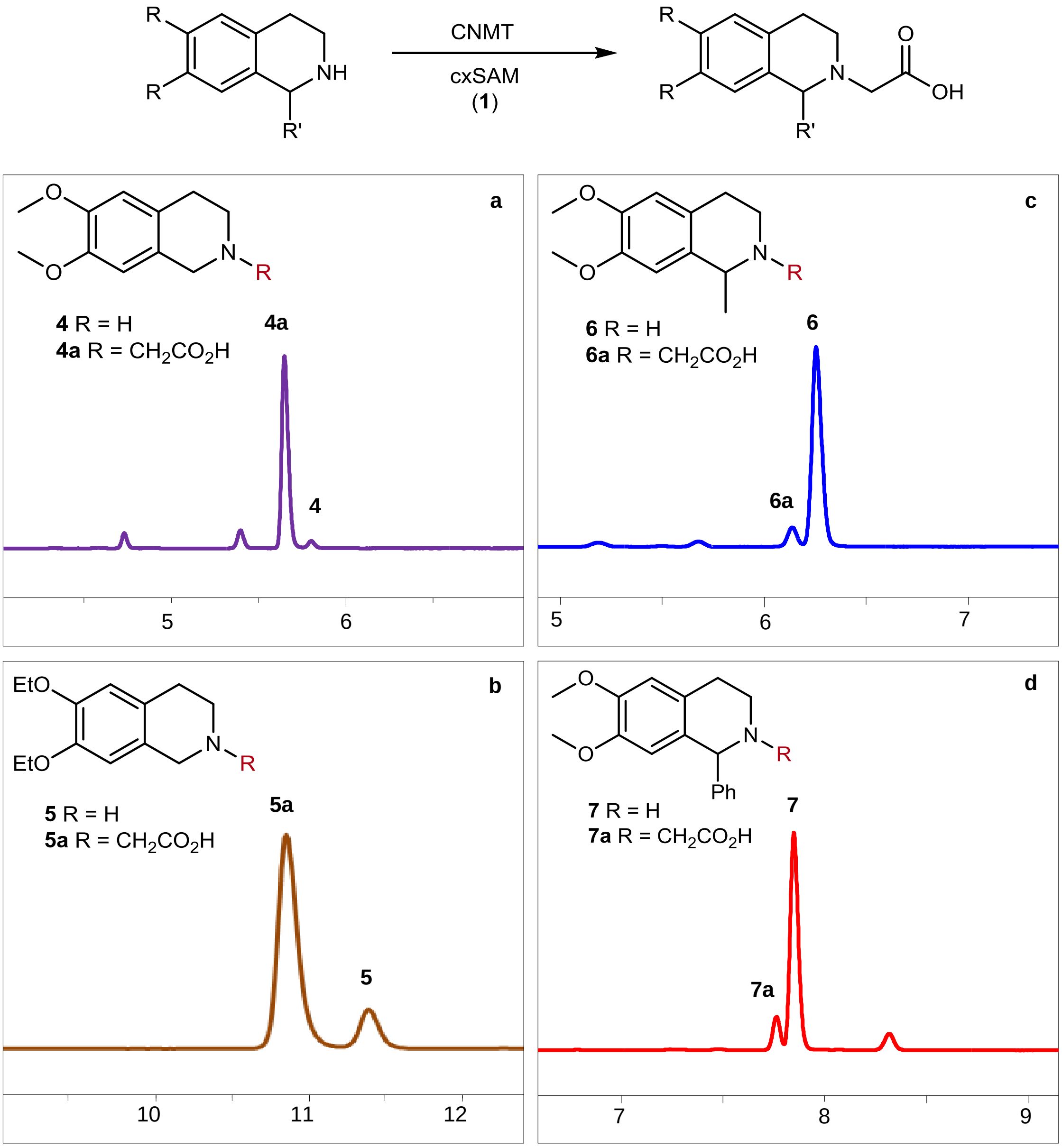

Figure S2. C18 HPLC analysis of assays of WT CNMT and synthetic cxSAM (**1**) with: (**a**) substrate **4** (purple); (**b**) substrate **5** (brown); (**c**) substrate **6** (blue); and (**d**) substrate **7** (red). CNMT (0.2 mM) was first incubated with substrate **4**-**7** (0.2 mM) in potassium phosphate buffer (100 mM, pH 7) at 37 ˚C for 1 hour. The mixture was then washed with buffer using a 30,000 MW centrifugal concentrator. Conversion assays were then conducted using synthetic cxSAM (**1**)(1.6 mM), substrate **4**-**7** (0.25 mM) and CNMT (100 µM) in buffer, incubated at 37 ˚C for 5 hours.

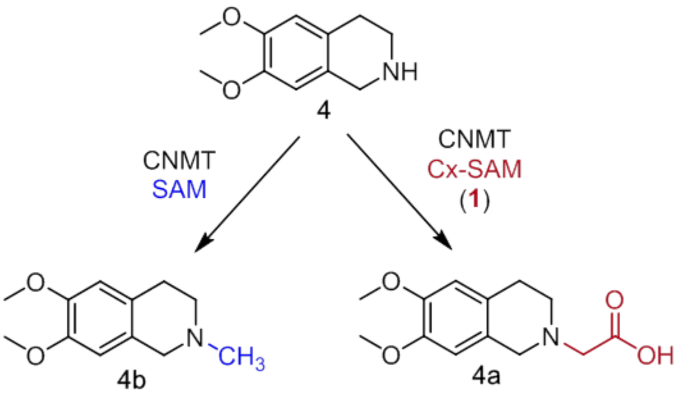

	Relative Activity to WT	
CNMT Mutant	SAM	cxSAM
WT	100	100
Y81R	13 ± 1	115 ± 1
Y81K	21 ± 1	78 ± 1
Y81F	44 ± 1	99 ± 1
Y81H	14 ± 1	78 ± 1

Figure S3. The relative activity of CNMT mutant enzymes against WT CNMT with Substrate **4** and SAM or cxSAM. CNMT (0.2 mM) was first incubated with substrate **5** (0.2 mM) in potassium phosphate buffer (100 mM, pH 7) at 37 ˚C for 1 hour. The mixture was then washed with buffer using a 30,000 MW centrifugal concentrator. Conversion assays were then conducted using synthetic cxSAM (**1**)(1.6 mM), substrate **4** (0.25 mM) and CNMT (100 μM) in buffer incubated at 37 ˚C for 17 hours. Assays were done in triplicate and standard error calculated.

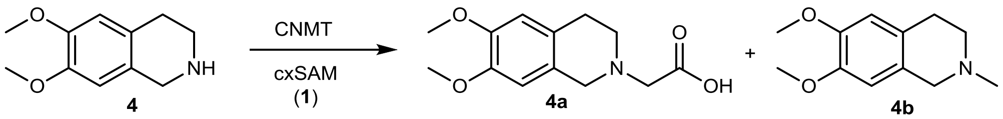

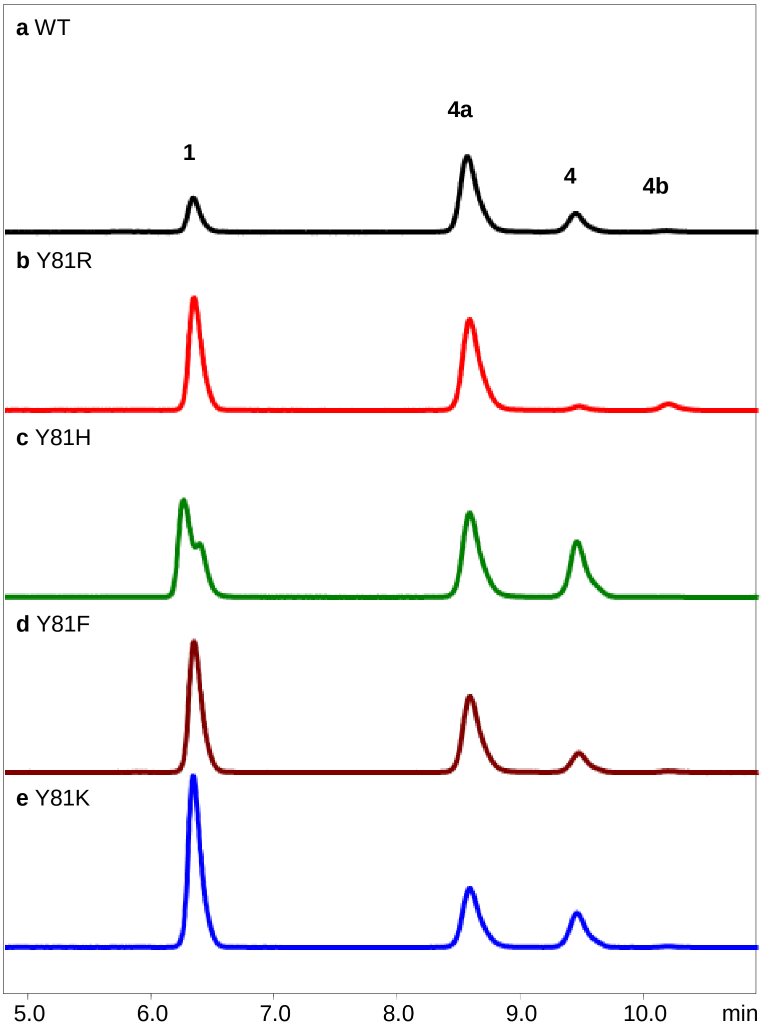

Figure S4. C18 RP HPLC analysis of enzyme activity assays of substrate **4** with: (**a**) WT CNMT (black); (**b**) CNMT Y81R (red); (**c**) CNMT Y81H (green); (**d**) CNMT Y81F (brown); and (**e**) CNMT Y81K (blue). CNMT (0.2 mM) was first incubated with substrate **5** (0.2 mM) in potassium phosphate buffer (100 mM, pH 7) at 37 ˚C for 1 hour. The mixture was then washed with buffer using a 30,000 MW centrifugal concentrator. Conversion assays were then conducted using synthetic cxSAM **1** (1.6 mM), substrate **4** (0.25 mM) and CNMT (100 μM) in buffer incubated at 37 ˚C for 17 hours.

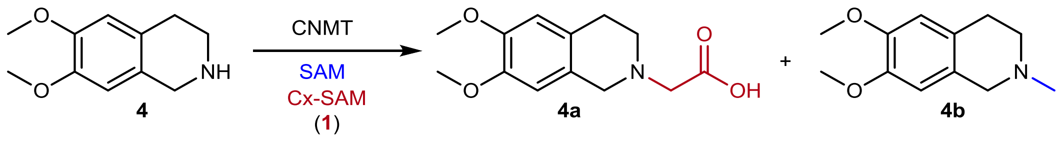

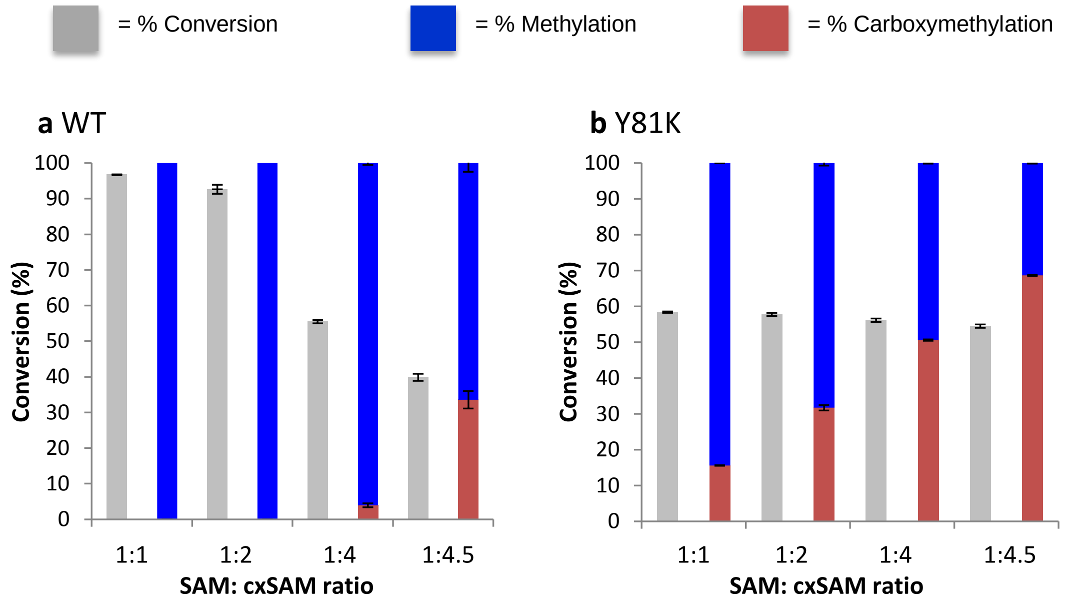

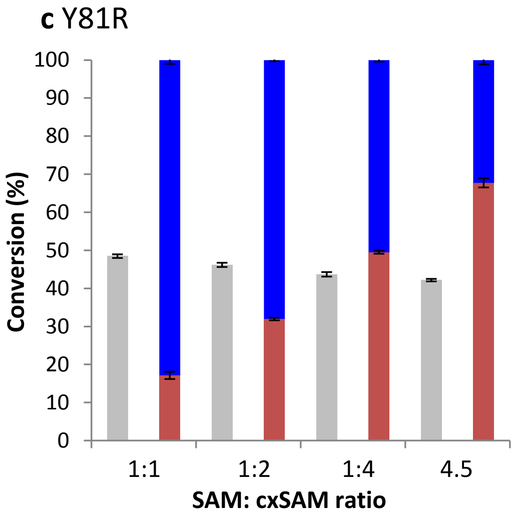

	Carboxymethylation (%)		
Ratio	WT	Y91K	Y81R
1:1	0 ± 1	16 ± 1	17 ± 1
1:2	0 ± 1	32 ± 1	32 ± 1
1:4	4 ± 1	51 ± 1	49 ± 1
1:4.5	33 ± 2	69 ± 1	68 ± 1

Figure S5. To further assess cofactor selectivity, the Y81R and Y81K mutants were compared with the WT CNMT in assays with varying ratios of SAM:cxSAM (1:1, 1:2, 1:4, 1:4.5 at 1 mM SAM and 1-9 mM cxSAM) and THIQ substrate **4** over a 5 hour incubation period. (**a**) WT CNMT (100 µM); (**b**) CNMT Y81R (100 µM); and (**c**) CNMT Y81K (100 µM) in potassium phosphate buffer (100 mM, 160 mM NaCl, pH 7) conducted at 37 ˚C, with constant agitation (800 rpm) for 5 hours. Assays were done in triplicate and standard error calculated. As expected both mutants (Y81R/K) displayed significantly improved cofactor selectivity for cxSAM, with 0% carboxymethylation observed with the WT enzyme at both 1:1 and 1:2 ratios of SAM:cxSAM, compared to 15-17% and *ca.* 32 % with both mutants at 1:1 and 1:2 (SAM:cxSAM), respectively. Upon increasing the ratio to 1:4 (SAM:cxSAM), Y81R/K both displayed a 49-50% carboxymethylation, compared to only 4% with WT.

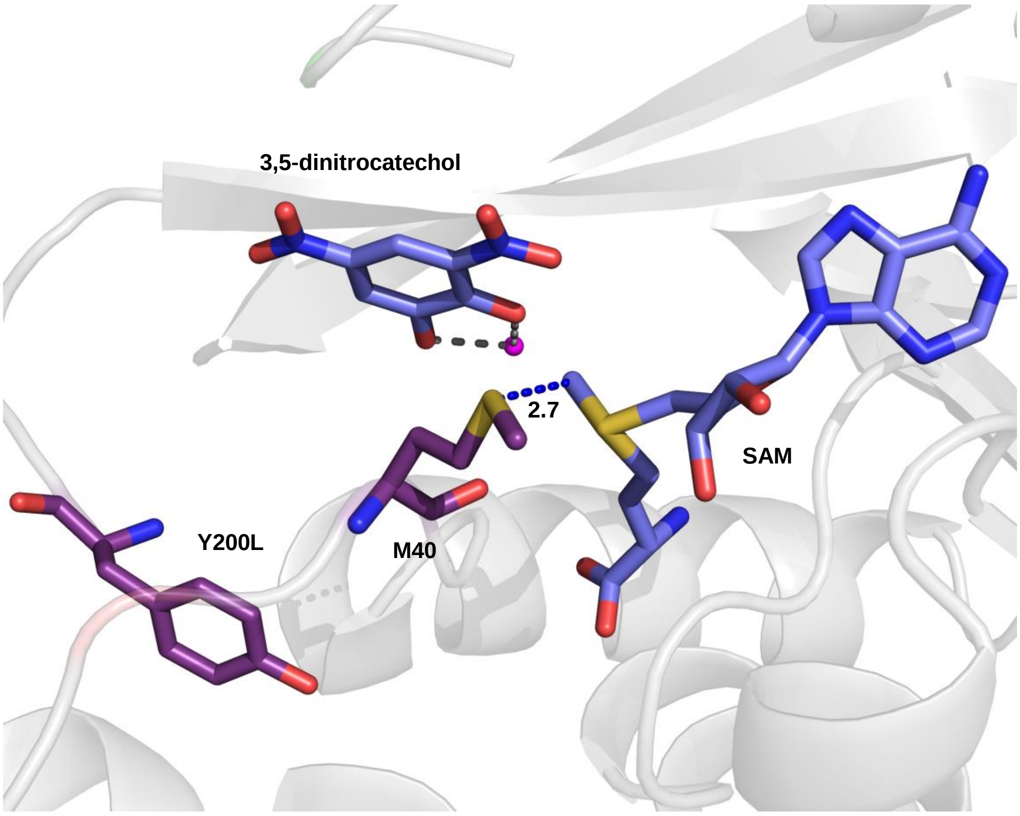

Figure S6. COMT (PDB: 5FHQ) active site with inhibitor 3,5-dinitrocatechol and SAM cofactor bound. The highlighted M40 was chosen as the site for point mutation due to its close (2.7 Å) proximity to the cofactor.

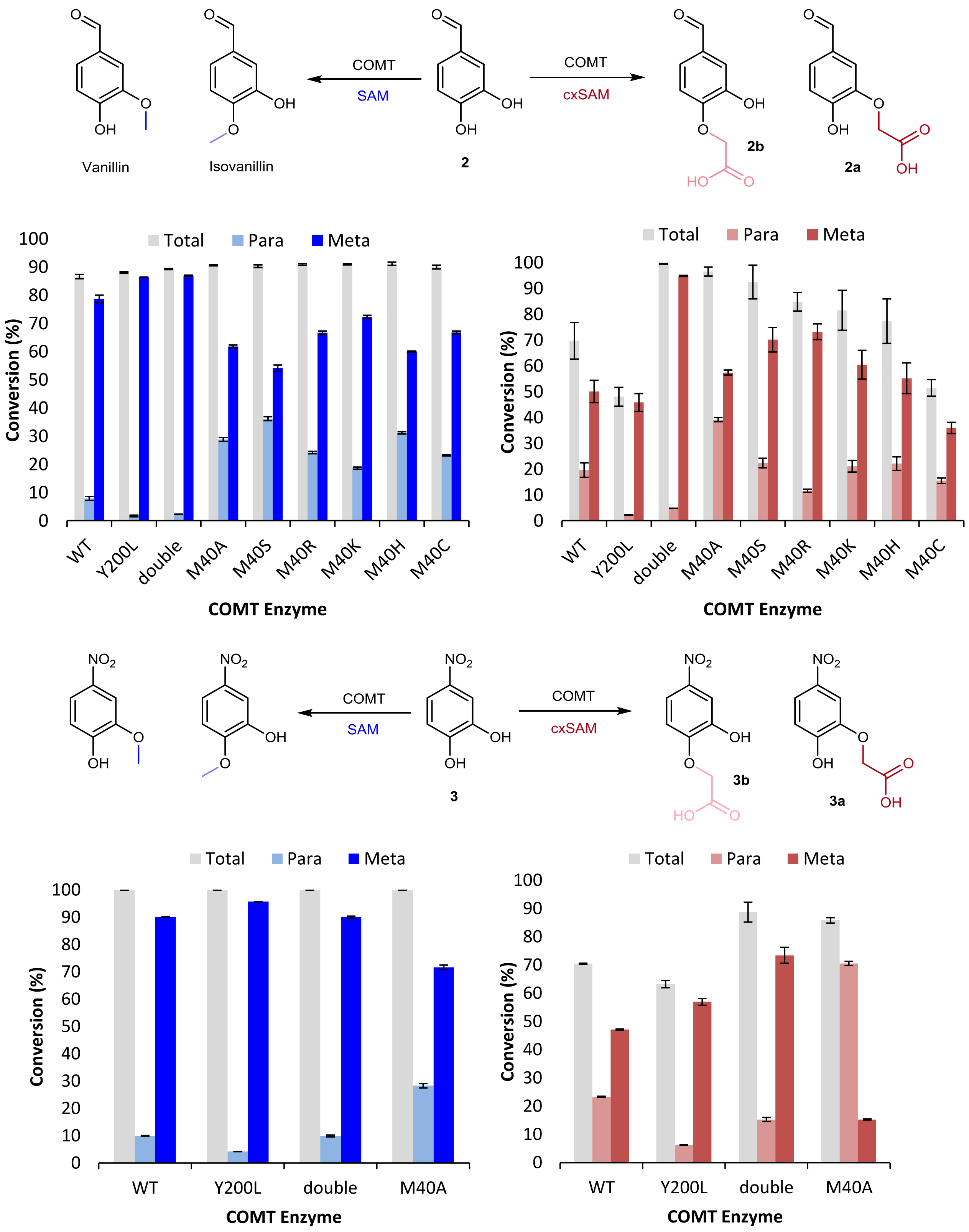

Figure S7. C18 RP HPLC analysis of enzyme activity assays of **a)** substrate **2** and **b)** substrate **3**. Assays of COMT M40 mutant enzymes with SAM (left) and cxSAM (right) over 17 hours. Assays were conducted using synthetic cxSAM **1** (1.6 mM) or SAM (Sigma)(1 mM), substrate **2** or **3** (0.25 mM), COMT (100 µM), $MgCl_2$ (6 mM), DTT (1 mM) in potassium phosphate buffer (50 mM, pH 7.4), incubated at 37 ˚C. Assays were done in triplicate and standard error calculated.

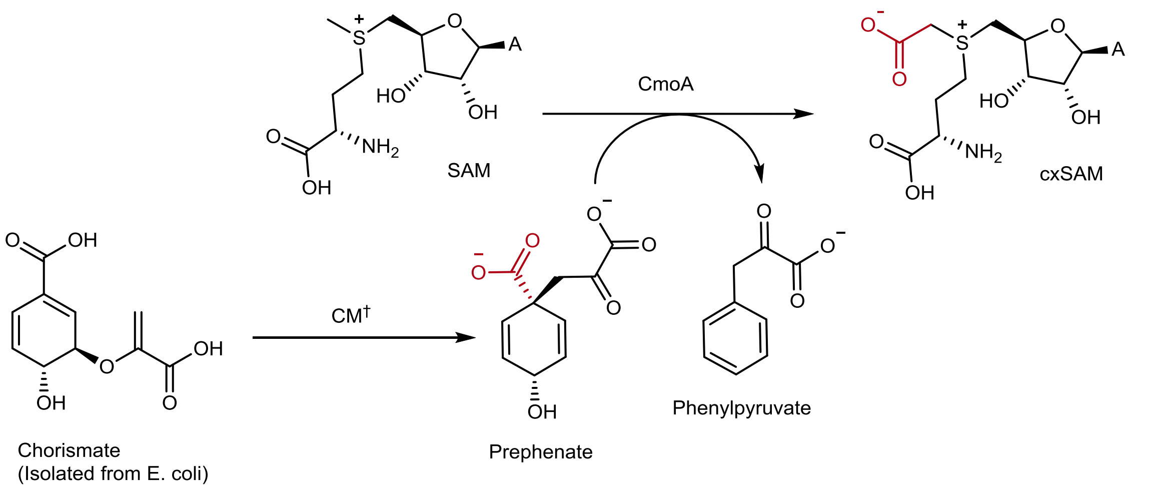

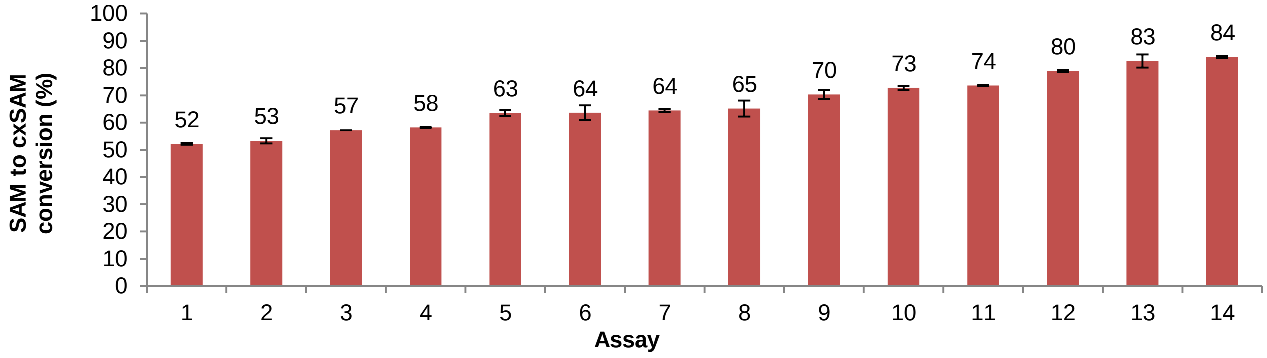

Assay	Conditions (5 h)	Chorismate (mM)	CM (μM)	CmoA (μM)	SAM (mM)
1	Chorismate added in 2 additions: t=0, t=1	2	1	100	0.5
2	Chorismate added in 2 additions: t=0, t=1	2	0	100	0.5
3		2	0.5	200	0.5
4		2	0	100	0.5
5		4	1	100	0.5
6	Chorismate added in 4 additions: t=0, t=1, t=2, t=3	4	0	100	0.5
7		4	0	100	0.5
8	Chorismate added in 4 additions: t=0, t=1, t=2, t=3	4	1	100	0.5
9		6	0	100	0.5
10	Additional CmoA added at t = 2.5h	2	0	200	0.5
11		4	0	200	0.5
12		6	0	200	0.5
13	Additional CmoA added at t = 2.5h	4	0	200	0.5
14	Additional CmoA added at t = 2.5h	6	0	200	0.5

Figure S8. CM-CmoA tandem assay optimisation showing the % conversion with conditions as shown within the table. Assays were done in triplicate and standard error calculated.

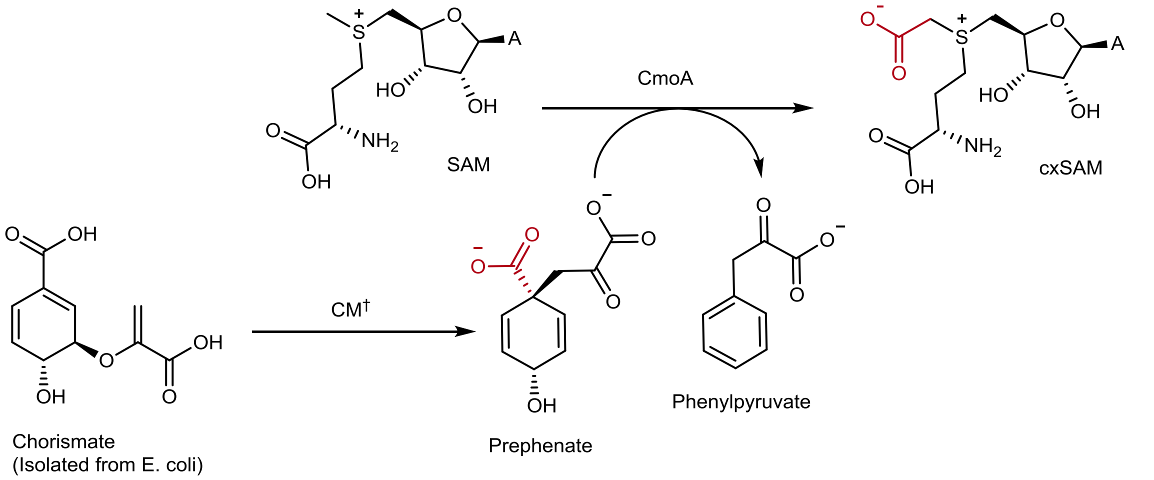

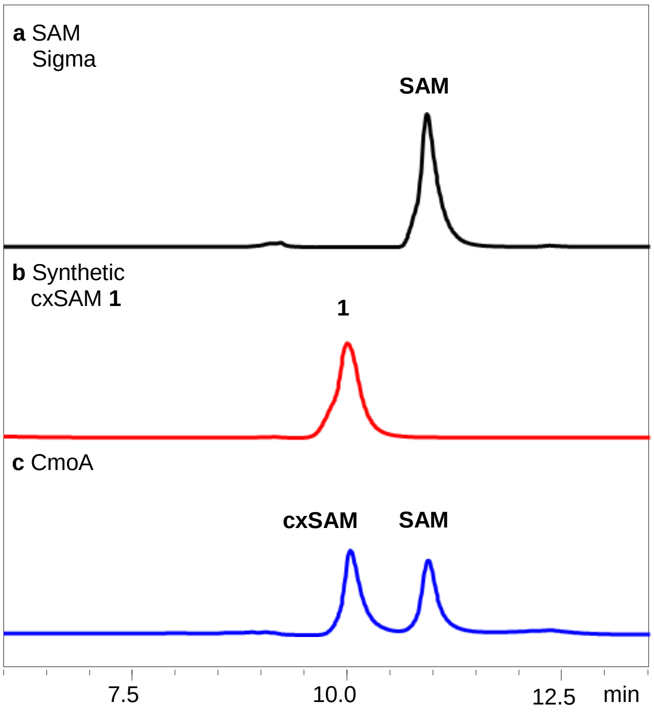

Figure S9. HILIC-HPLC analysis from the CmoA catalysed conversion of SAM to cxSAM after 3 hours incubation. (**a**) Commercial SAM (Sigma)(black); (**b**) synthetic cxSAM **1** (red); and (**c**) CmoA assay with SAM (Sigma)(blue). The assay was conducted using chorismate (6 mM), SAM (0.5 mM) and CmoA (200 µM) in potassium phosphate buffer (100 mM, pH 7) conducted at 37 ˚C, with constant agitation (800 rpm), for 3 hours.

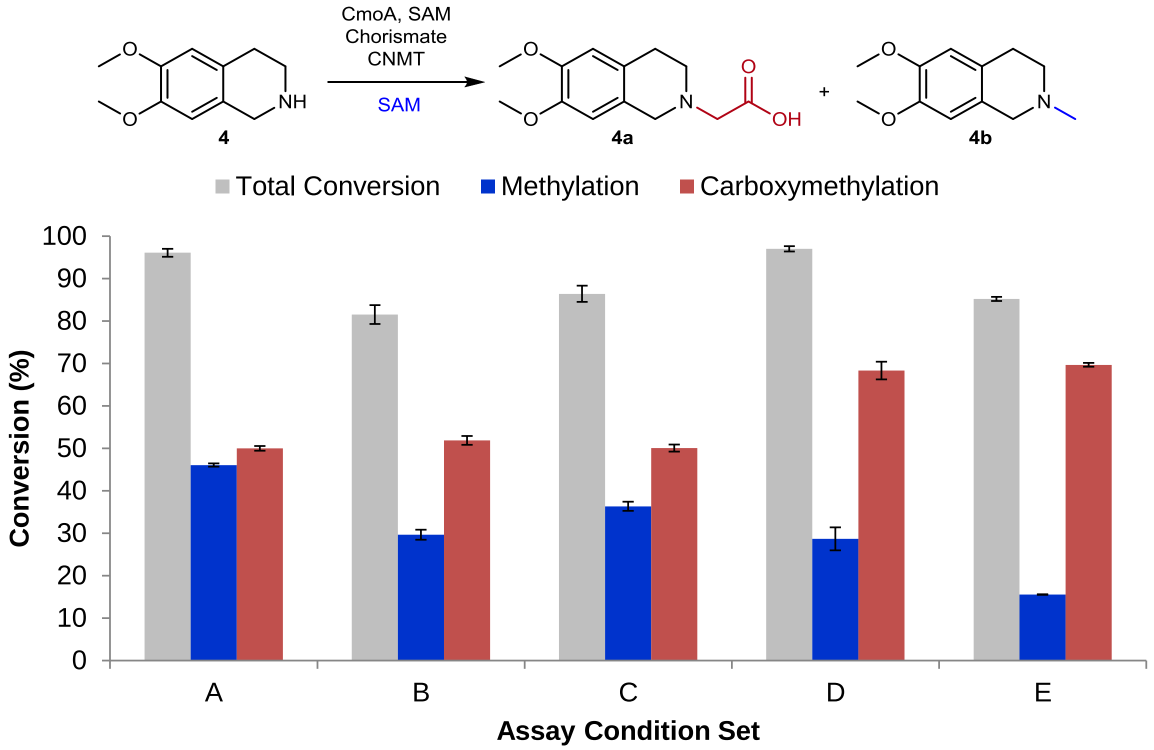

		Part 1: 37 ˚C, 800 rpm, 5 hours/h, pH 8			Part 2: 37 ˚C, 800 rpm, 17 h	
Assay	CNMT	Chorismate (mM)	SAM (mM)	CmoA (µM)	**4** (mM)	CNMT (µM)
A	WT	4	0.5	100	0.25	100
B	Y81R	4	0.5	100	0.25	100
C	Y81R	6	0.5	400	0.25	100
D	Y81R	6	0.5	400	0.1	100
E	Y81R	6	0.5	200	0.1	100*

Figure S10. Optimisation of the CmoA-CNMT tandem coupled assay. The assay conditions for the tandem coupled assays A–E with **4** are shown in the table. * Pre-incubated with substrate **5**. Assays were done in triplicate and standard error calculated.

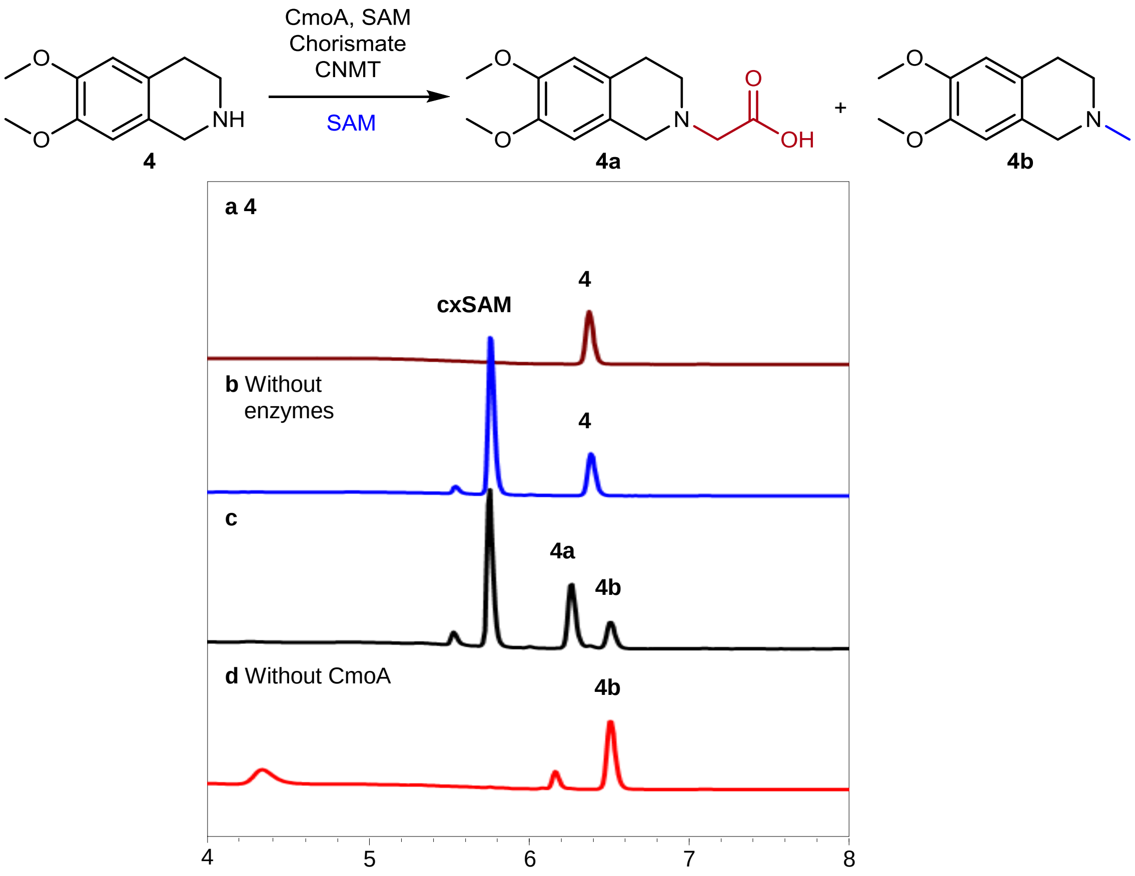

Figure S11. C18 RP HPLC analysis of WT CNMT and CmoA coupled assays with **4** and SAM; (**a**) Commercial **4** (Sigma)(brown); (**b**) coupled assay without CmoA and CNMT (blue); (**c**) CmoA and CNMT assay with SAM (Sigma) and **4** (black); and (**d**) coupled assay without CmoA (red). The assay was conducted using SAM (0.5 mM), chorismate (6 mM) and CmoA (200 µM) in potassium phosphate buffer (100 mM, pH 7) at 37 ˚C for 5 hours. Following this, **4** (0.1 mM) and CNMT (100 µM) were added and further incubated at 37 ˚C for 17 hours.

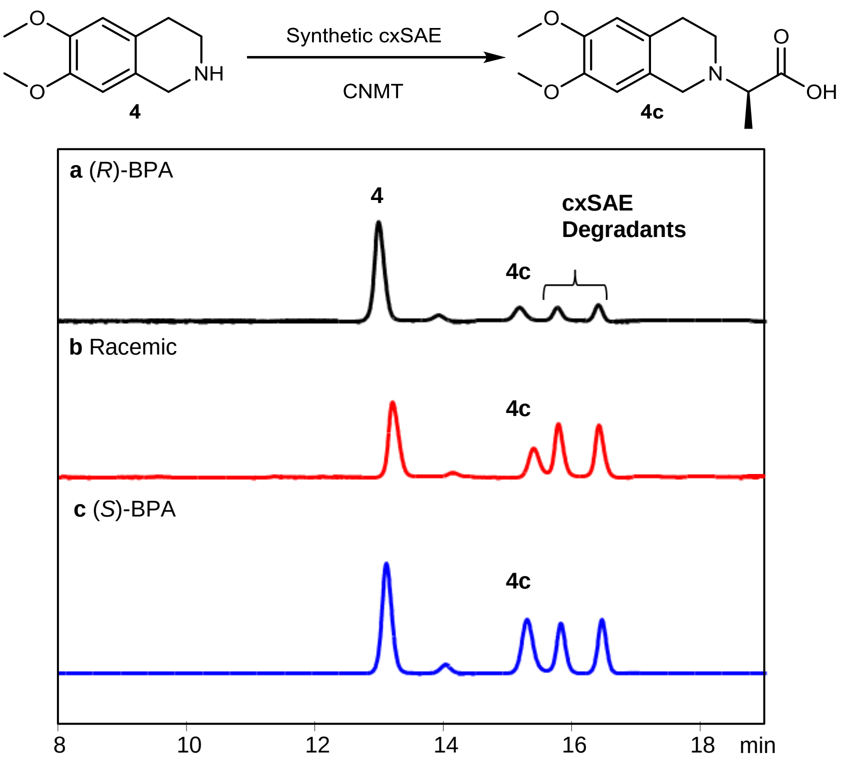

Figure S12. C18 RP HPLC analysis of WT CNMT activity assays with **4** and synthetic cxSAE; (**a**) (*R*)-BPA derived cxSAE (black); (**b**) racemic BPA derived cxSAE (red); and (**c**) (*S*)-BPA derived cxSAE (blue). CNMT (0.2 mM) was first incubated with substrate **5** (0.2 mM) in potassium phosphate buffer (100 mM, pH 7) for 1 hour at 37 ˚C. The mixture was then washed with buffer using a 30,000 MW centrifugal concentrator. Conversion assays were then conducted using synthetic cxSAE (**8**, **9** or **10**)(1.6 mM), substrate **4** (0.25 mM) and CNMT (100 µM) in buffer incubated at 37 ˚C for 17 hours.

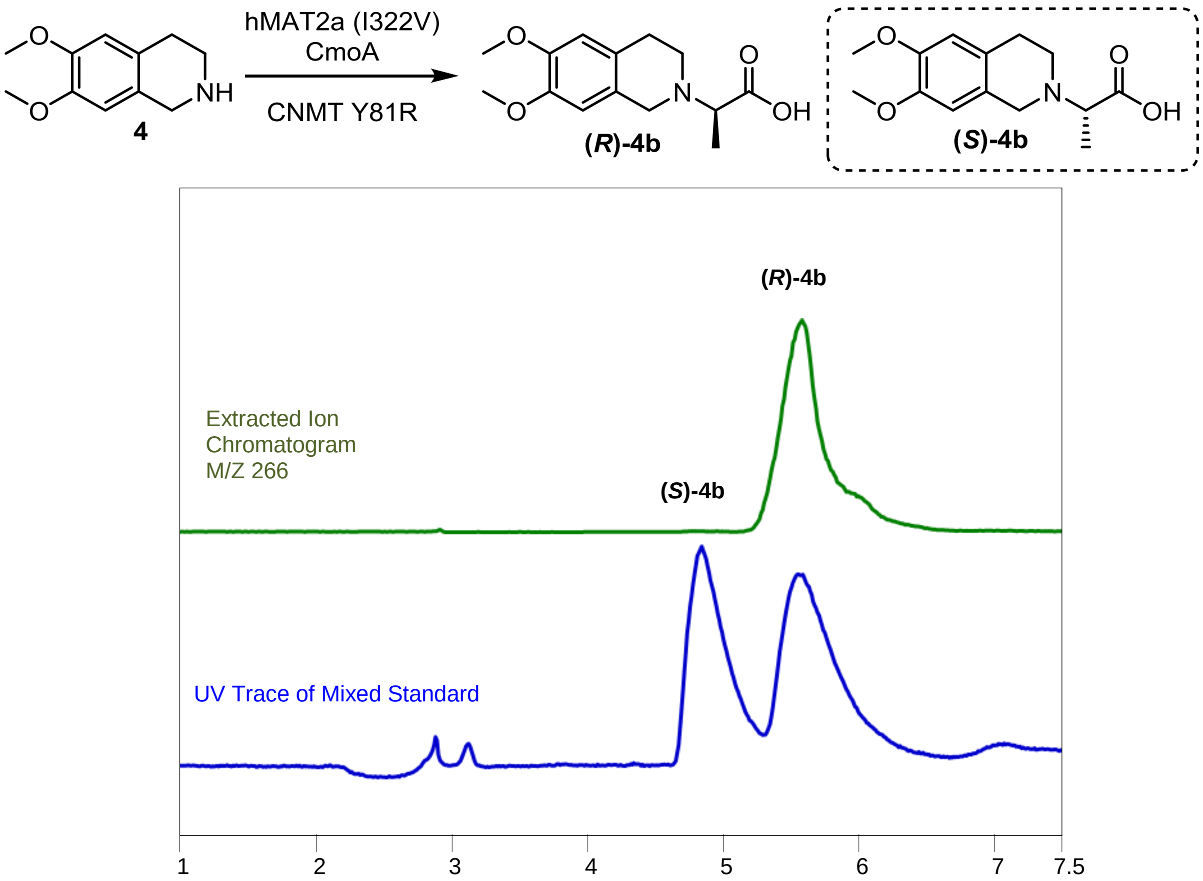

Figure S13. Chiral-HPLC-MS analysis of hMAT, CmoA and Y81R CNMT combined assay to produce ***(R)*-4b**. Combined assay extracted ion chromatogram of **4b** $[M+H]^+$ m/z 266 (green) and a combined product standard (blue). This shows that though the combined assay results in interfering peaks within the region of interest, only the ***(R)*-4b** isomer is present. The assay was conducted using ATP (0.5 mM), DTT (1 mM), L-ethionine (0.5 mM), hMAT2a I322V (68 µM) and $MgCl_2$ (5 mM) in potassium phosphate buffer (100 mM, pH 7). The reaction mixture was incubated for 60 mins at 37 ˚C with constant agitation (850 rpm). Prephenate (1.25 mM) and CmoA (50 µM) were added and incubated for 240 mins. **4** (0.1 mM) and pre-incubated CNMT Y81R (100 µM) were then added and incubated at 30 ˚C with constant agitation (850 rpm) for 17 hours.

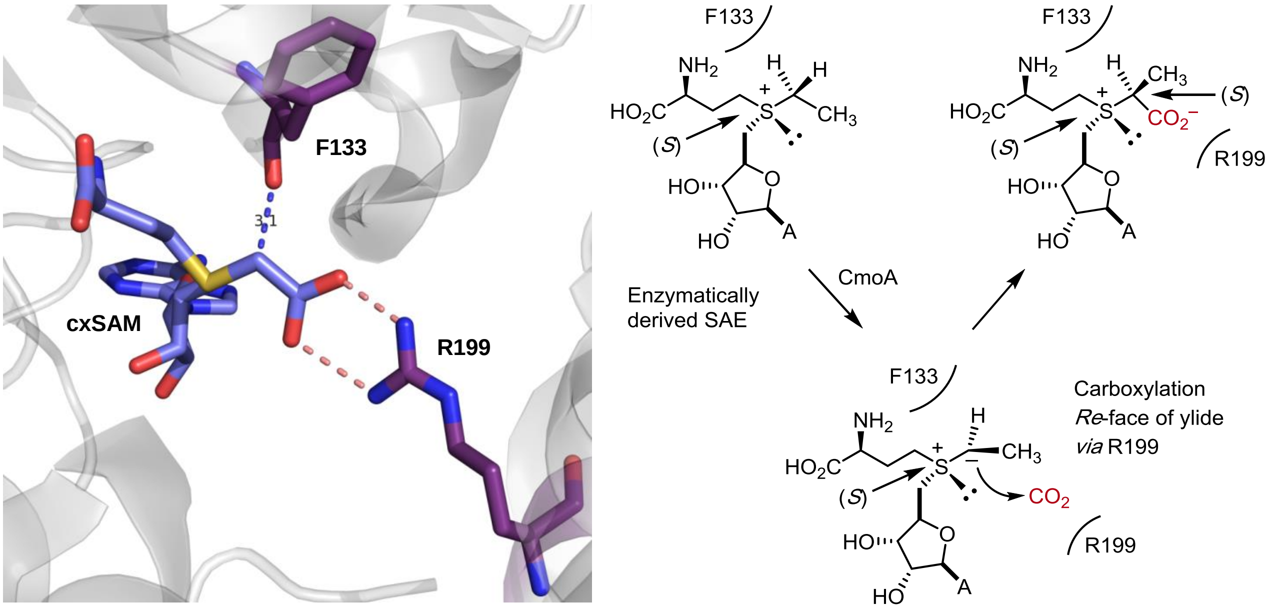

Figure S14. Structure of CmoA with CxSAM bound and predicted stereochemical course of enzymatic SAE carboxylation. Enzymatic SAE possesses the natural (*S*)-configured sulphonium stereocenter based on the known stereoselectivity of hMAT. In addition, from the published structure (PDB 4GEK) and models of the CmoA active site with prephenate bound,[19] we suggest that the ethyl substituent of (SAE) would be orientated away from the F133 residue due to their close proximity (3.1 Å) in the CmoA active site. Deprotonation of SAE would generate a sulphonium ylid that is predicted to be carboxylated from the *Re*-face, via the R199 residue (on the opposite side of the active site to F133), which results in the 1-(*S*)-carboxyethyl substituent as drawn.

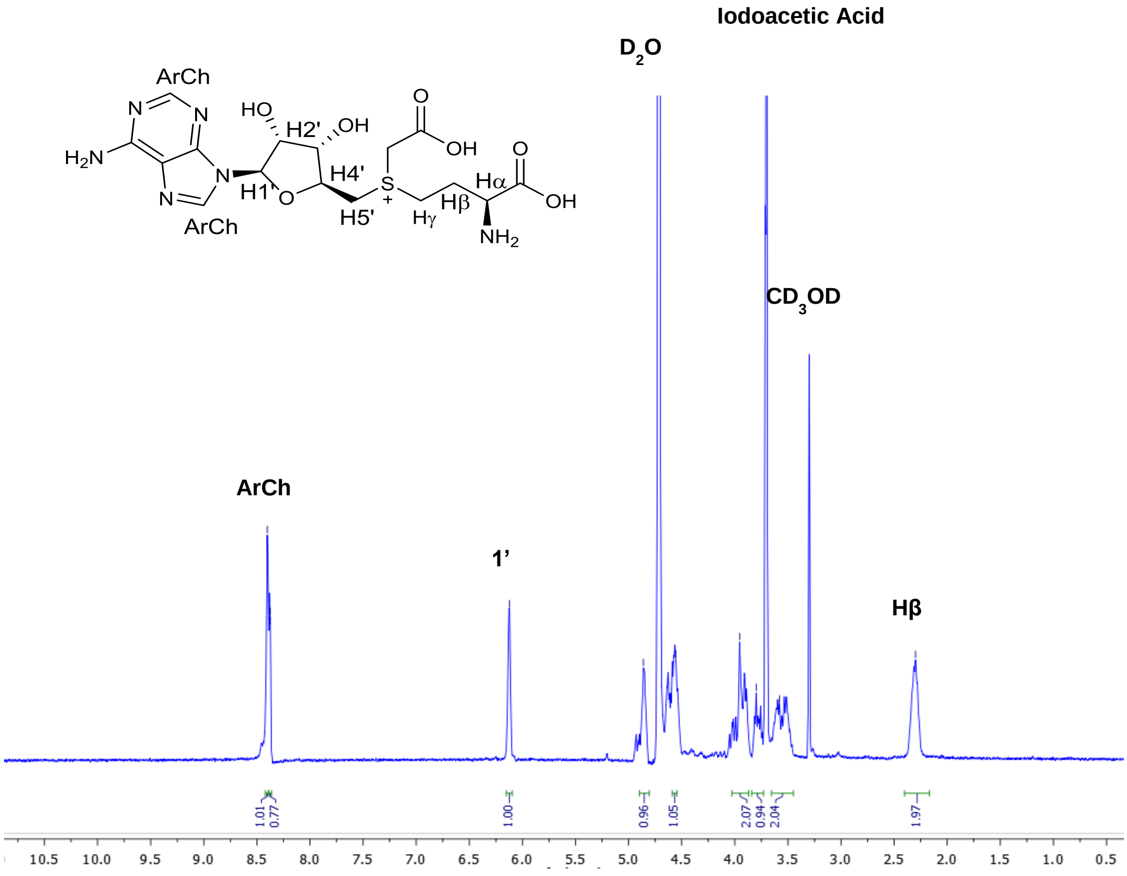

Figure S15. ^{1}H-NMR analysis of synthetic CxSAM **(1).**

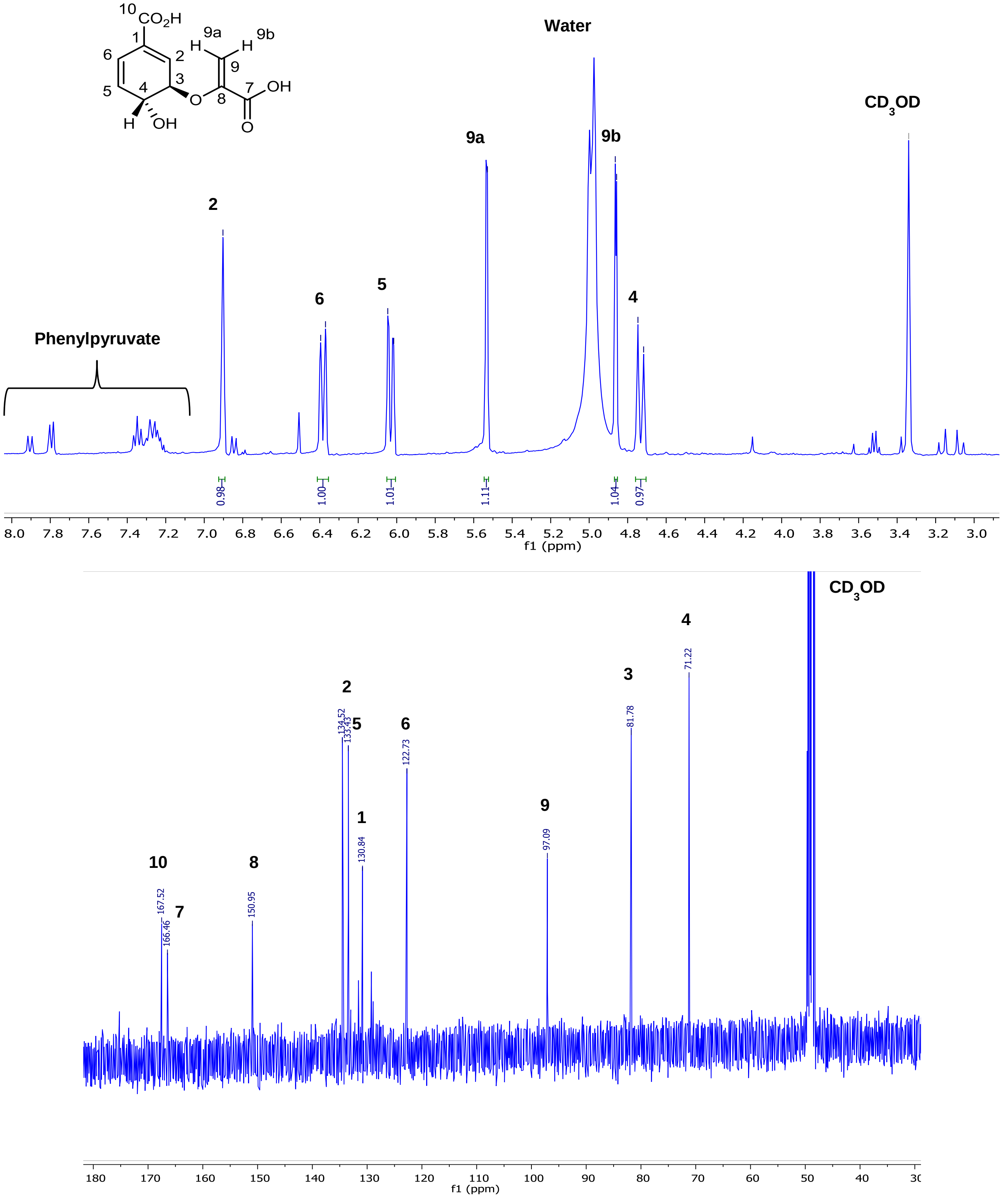

Figure S16. ^{1}H (top) and ^{13}C-NMR (bottom) analysis of the biosynthetic chorismate extracted from the *E..coli* KA12 strain.

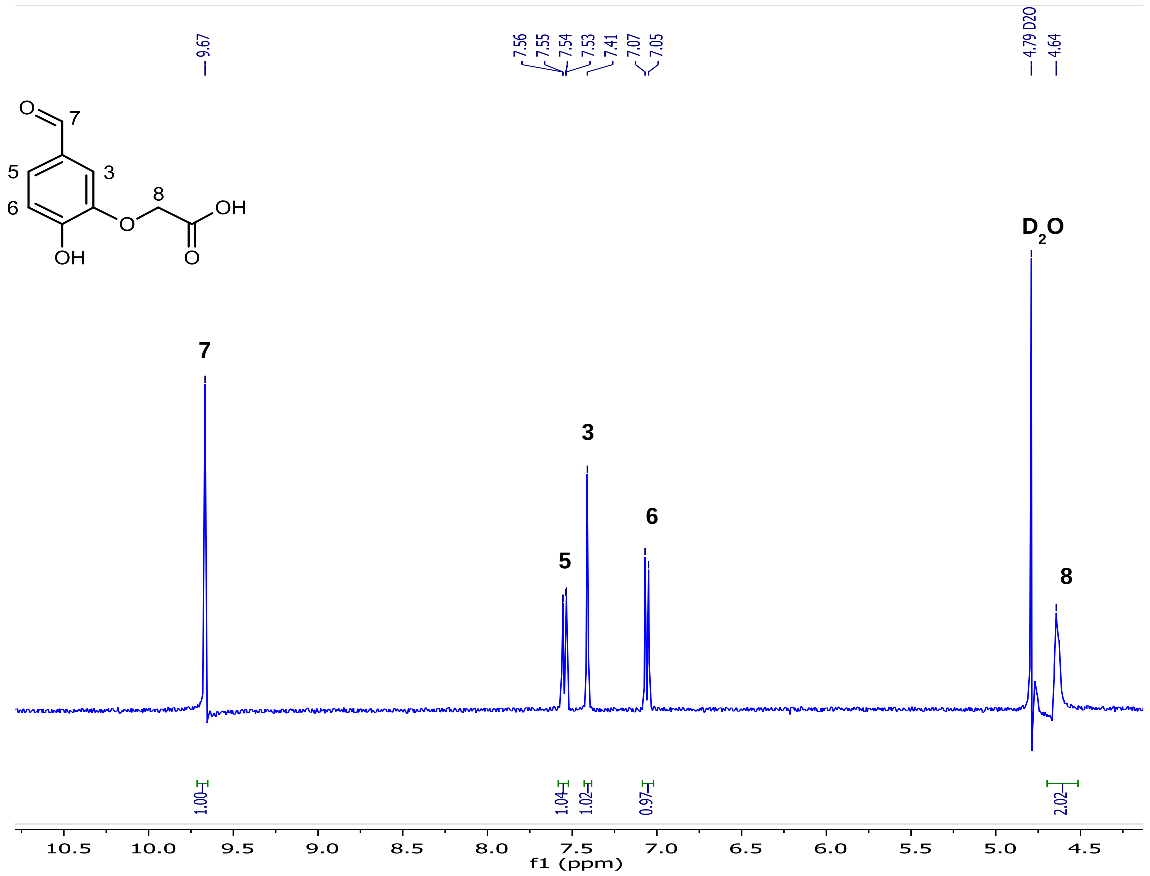

Figure S17. ^{1}H NMR analysis of biosynthetic *meta*- product: 2-(4-formyl-1-hydroxyphenoxy)acetic acid (**2a**).

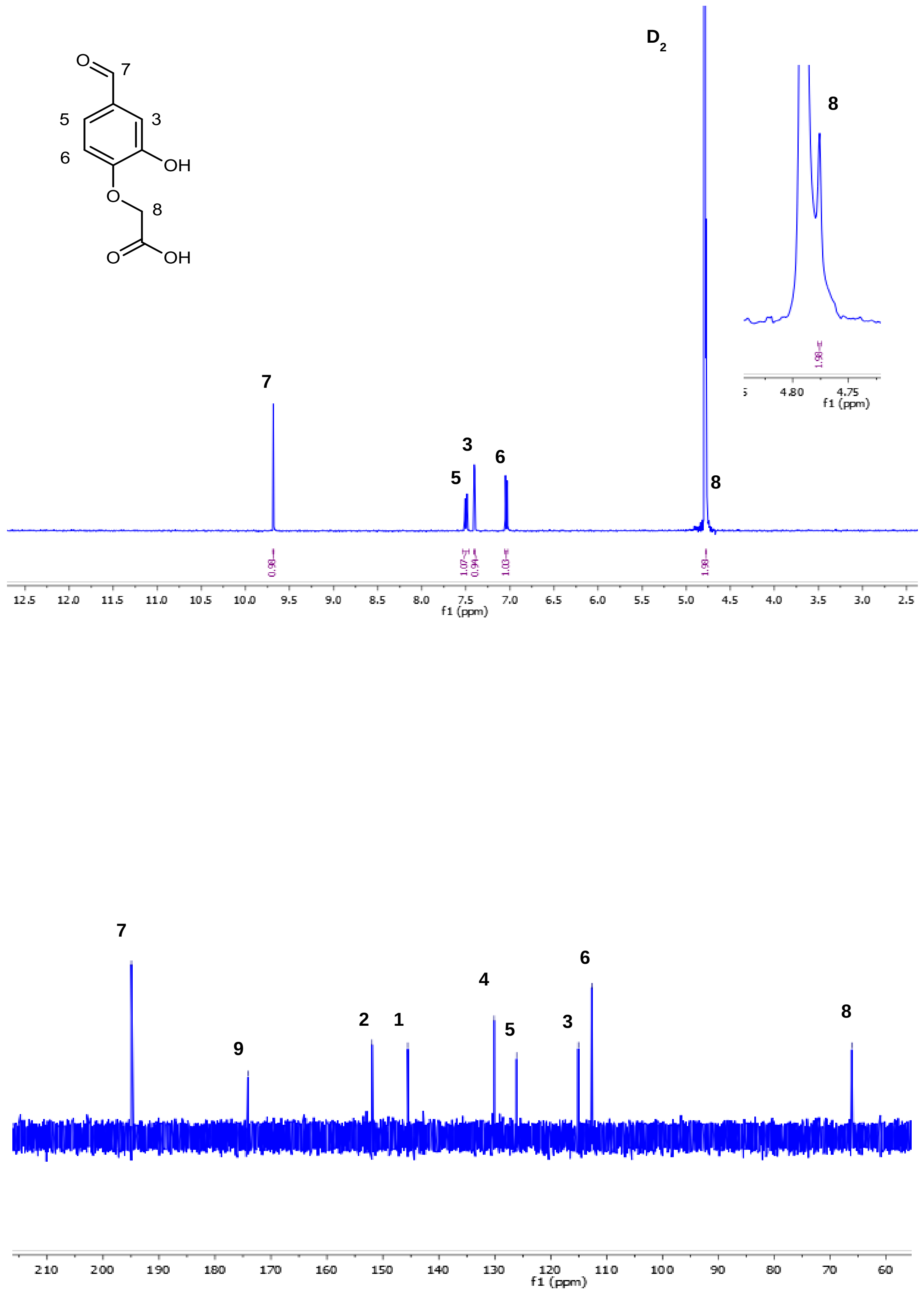

Figure S18. ^{1}H (top) and ^{13}C-NMR (bottom) analysis of synthetic *para*- product: 2-(4-formyl-2-hydroxyphenoxy)acetic acid (**2b**).

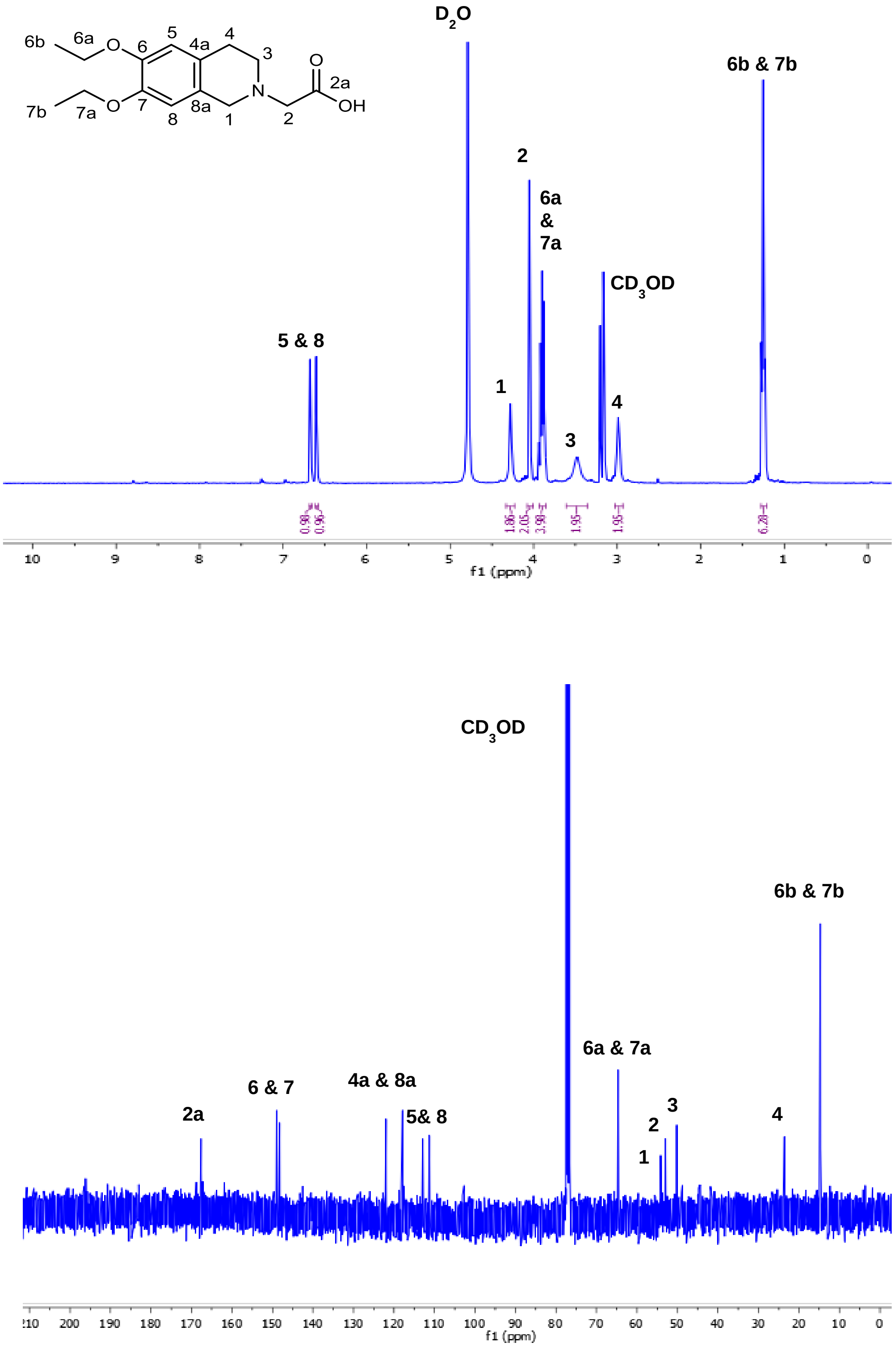

Figure S19. ^{1}H (top) and ^{13}C-NMR (bottom) analysis of synthetic *N*-carboxymethyl 6,7-diethoxy-1,2,3,4-tetrahydroisoquinoline (**5a**).

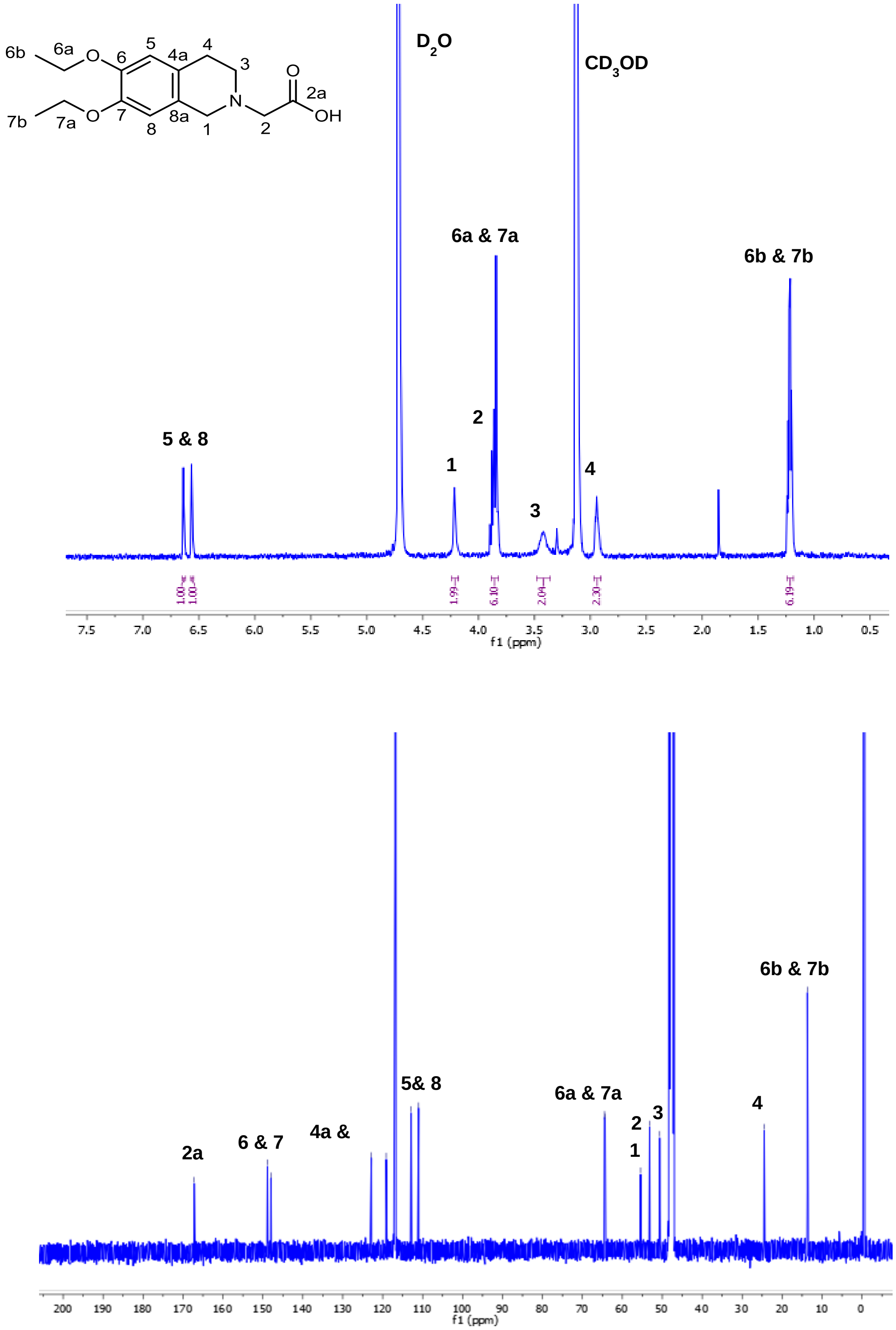

Figure S20. ^{1}H (top) and ^{13}C-NMR (bottom) analysis of enzymatic *N*-carboxymethyl 6,7-diethoxy-1,2,3,4-tetrahydroisoquinoline (**5a**).

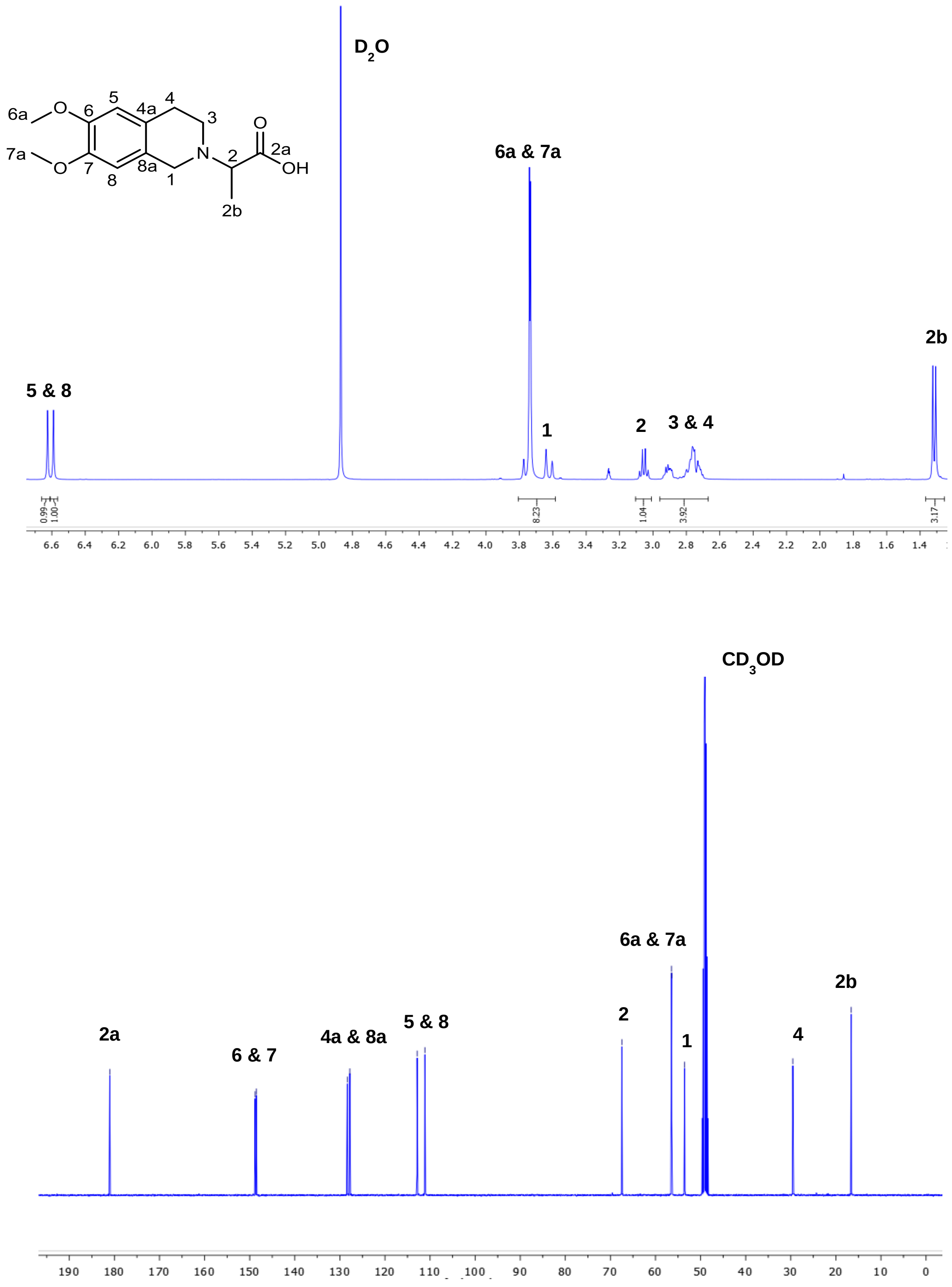

Figure S21. ^{1}H (top) and ^{13}C-NMR (bottom) analysis of synthetic (±)-*N*-carboxyethyl 6,7-dimethoxy-1,2,3,4-tetrahydroisoquinoline (**4**).

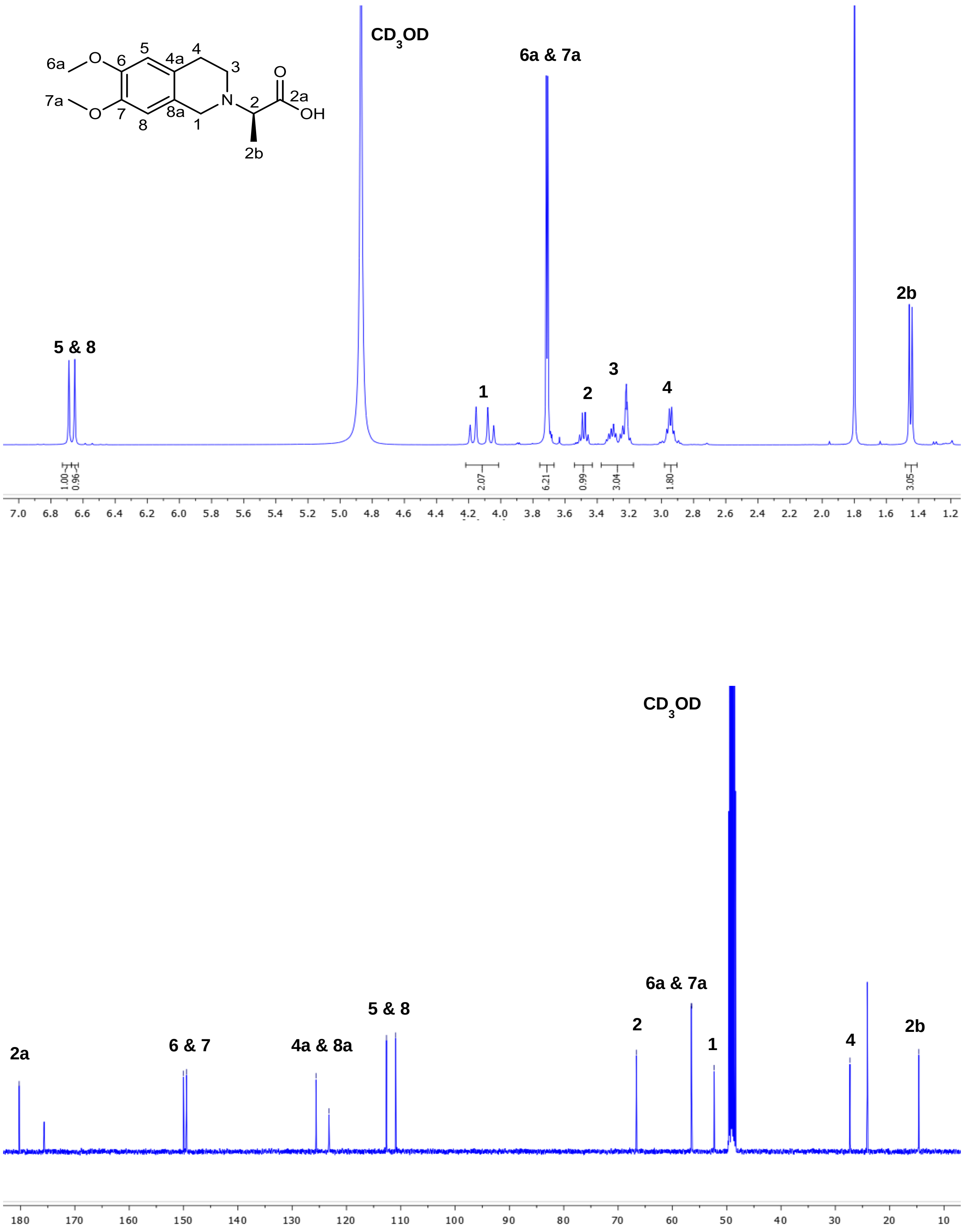

Figure S22. ^{1}H (top) and ^{13}C-NMR (bottom) analysis of synthetic (*R*)-*N*-carboxyethyl 6,7-dimethoxy-1,2,3,4-tetrahydroisoquinoline (**(*R*)-4b**).

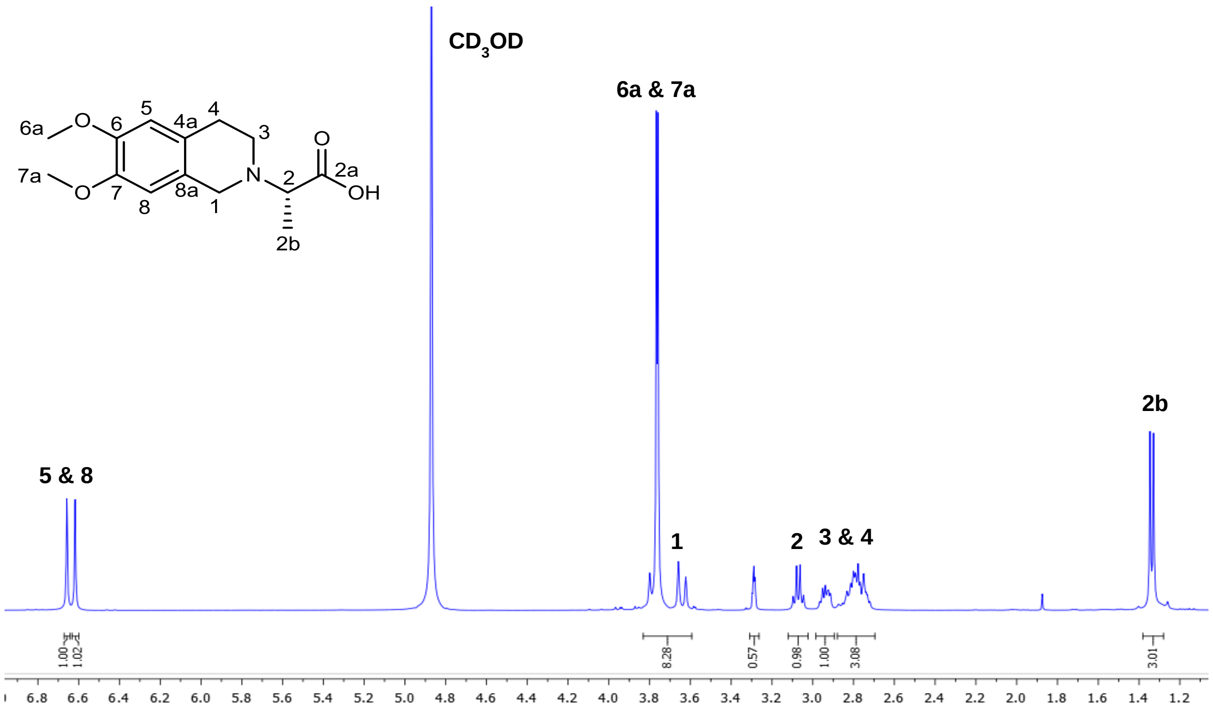

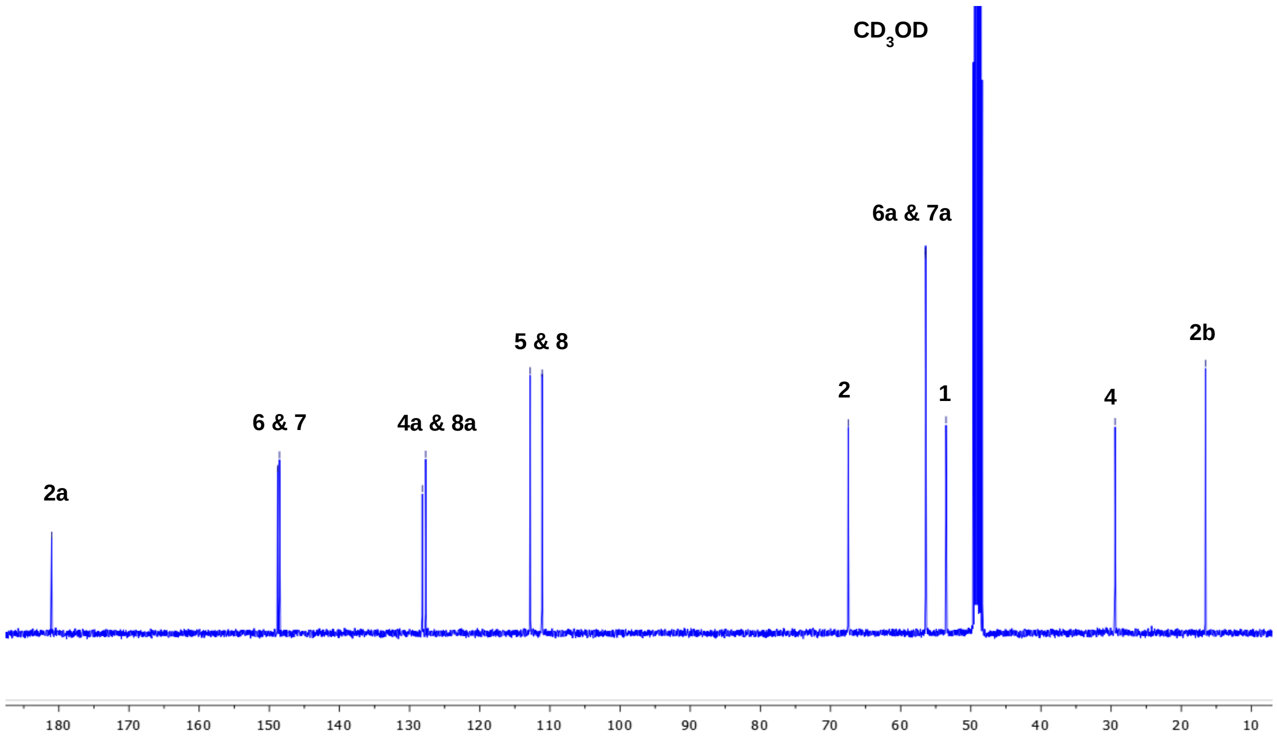

Figure S23. ^{1}H (top panel) and ^{13}C-NMR (bottom panel) analysis of synthetic (*S*)-*N*-carboxyethylated 6,7-dimethoxy-1,2,3,4-tetrahydroisoquinoline (**(*S*)-4b**).

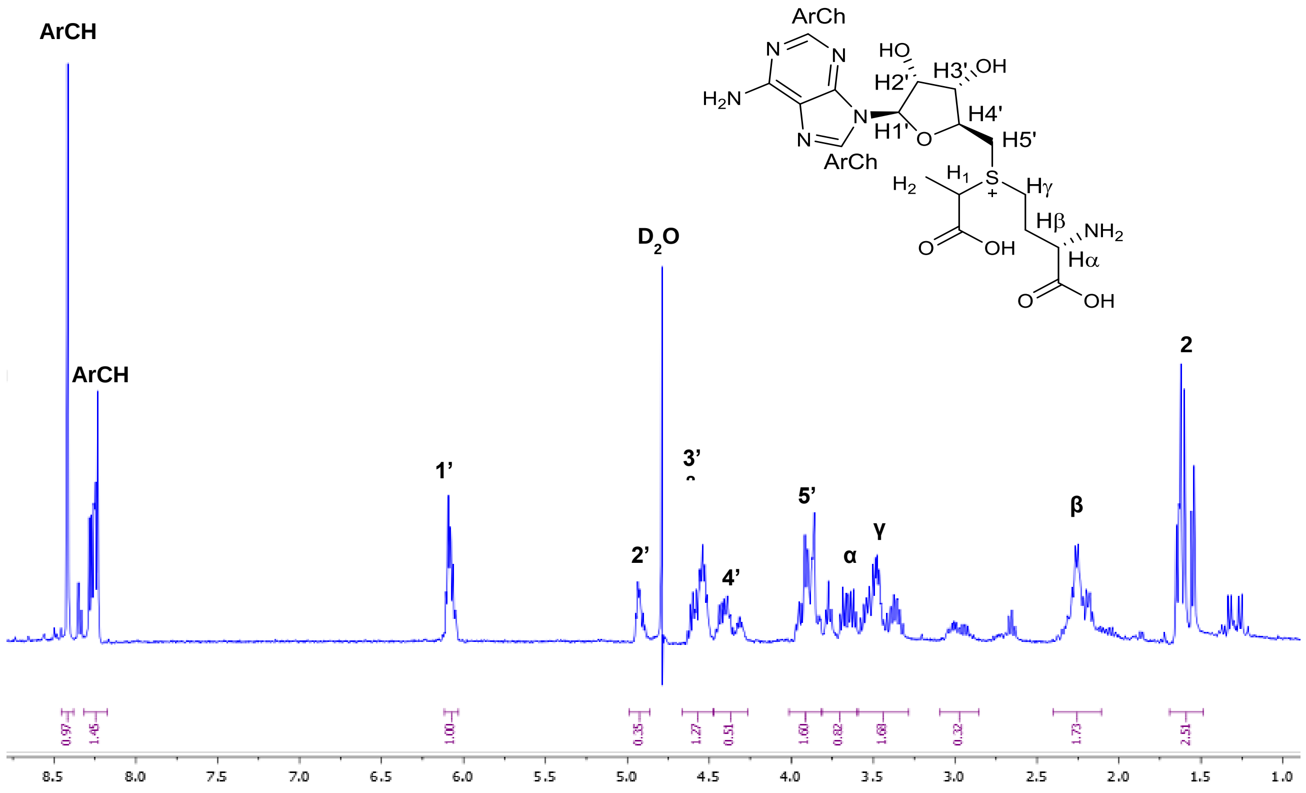

Figure S24. ^{1}H-NMR analysis of synthetic racemic cxSAE (**8**).

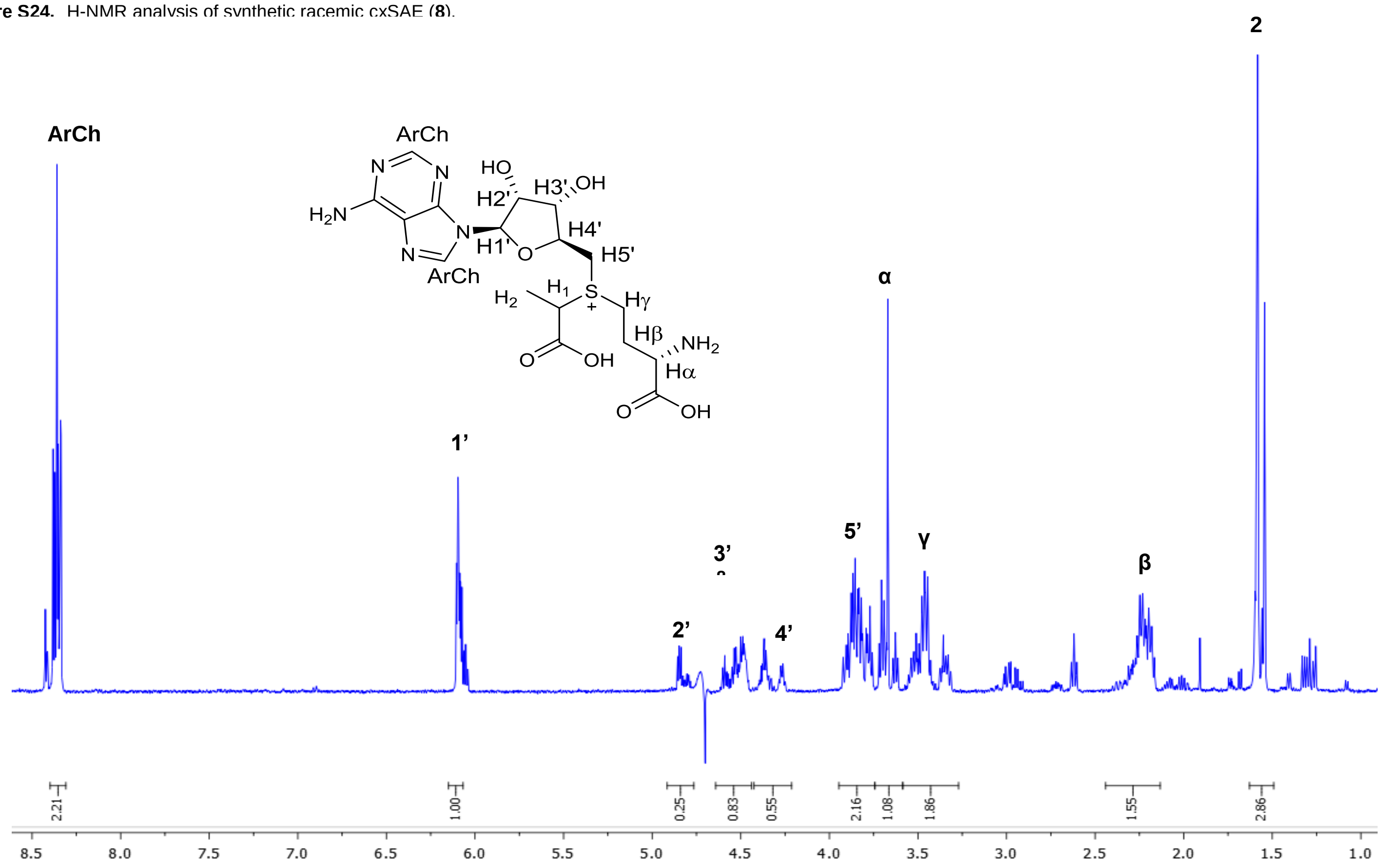

Figure S25. ^{1}H-NMR analysis of synthetic cxSAE (**9**).

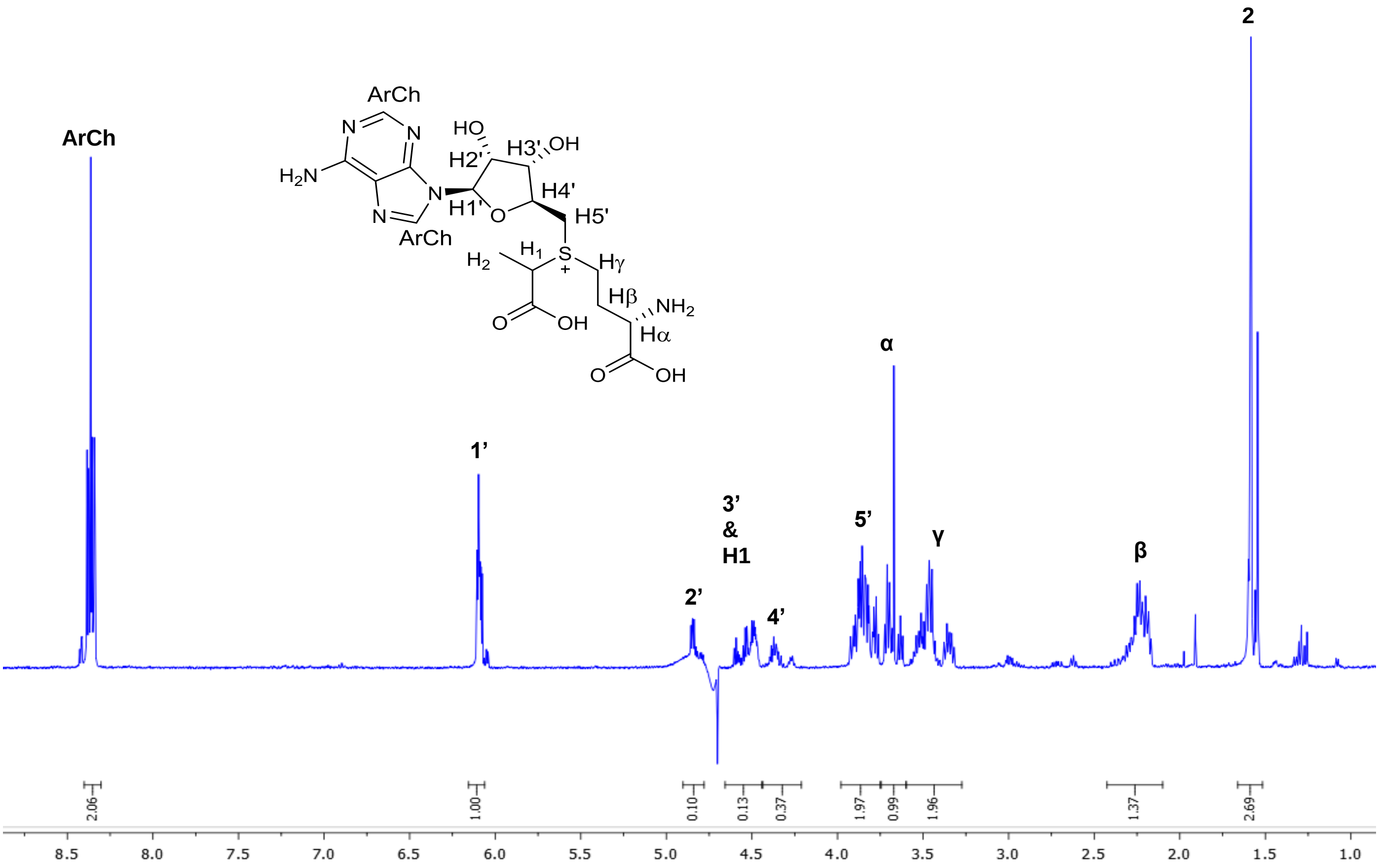

Figure S26. ^{1}H-NMR analysis of synthetic cxSAE (**10**).

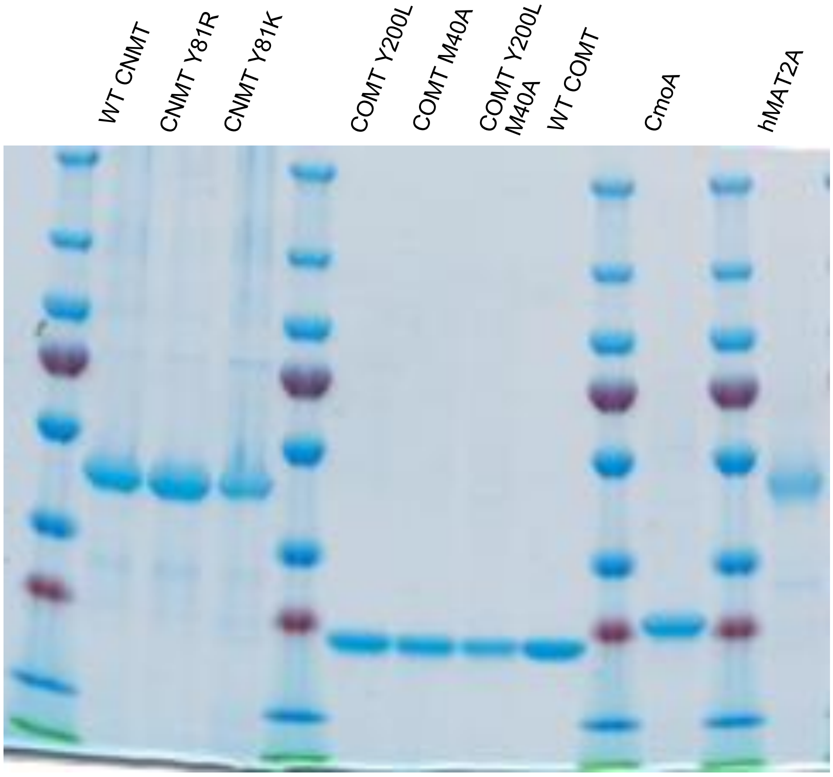

Figure S27. SDS page gel of purified proteins – PageRuler Plus Prestained Protein Ladder.

Supplementary Tables

	Name	Primer 5'-3'	Restriction Enzyme
**1**	CmoA F	AAAAAA<u>CATATG</u>TCTCACCGCGACACGC	*NdeI*
**2**	CmoA R	AAAAAA<u>CTCGAG</u>TCATGCAGCGTCCTCTGTGCTT	*XhoI*
**3**	CNMT Y81R F	GATAGCCAGCTGAGGGAAATTCCGATTG	
**4**	CNMT Y81R R	AATCGGAATTTCCCTCAGCTGGCTATCC	
**5**	CNMT Y81K F	GATAGCCAGCTGAAGGAAATTCCGATTG	
**6**	CNMT Y81K R	CAATCGGAATTTCCTTCAGCTGGCTATC	
**7**	CNMT Y81H F	ATAGCCAGCTGCATGAAATTCCG	
**8**	CNMT Y81H R	CGGAATTTCATGCAGCTGGCTAT	
**9**	CNMT Y81F F	GATAGCCAGCTGTTTGAAATTCCGATTG	
**10**	CNMT Y81F R	AATCGGAATTTCAAACAGCTGGCTATCC	
**11**	CNMT E204A F	TCTGGTGATCGCACTGTTTGAAC	
**12**	CNMT E204A R	GTTCAAACAGTGCGATCACCAGA	
**13**	CNMT E204K F	CGTATTCTGGTGATCAAGCTGTTTGAACATATG	
**14**	CNMT E204K R	CATATGTTCAAACAGCTTGATCACCAGAATACG	
**15**	CNMT E204S F	TTCTGGTGATCTCACTGTTTGAAC	
**16**	CNMT E204S R	GTTCAAACAGTGAGATCACCAGAA	
**17**	CNMT E207G F	CGAACTGTTTGGCCATATGAAAAAC	
**18**	CNMT E207G R	TTTTTCATATGGCCAAACAGTTCGA	
**19**	CNMT E207K F	GGTGATCGAACTGTTTAAGCATATGAAAAACTACG	
**20**	CNMT E207K R	CGTAGTTTTTCATATGCTTAAACAGTTCGATCACC	
**21**	CNMT E207R F	GGTGATCGAACTGTTTCGCCATATGAAAAACTAC	
**22**	CNMT E207R R	CGTAGTTTTTCATATGGCGAAACAGTTCGATCAC	
**23**	CNMT E207S F	GATCGAACTGTTTAGCCATATGAAAAAC	
**24**	CNMT E207S R	AGTTTTTCATATGGCTAAACAGTTCGAT	
**25**	COMT M40A R	TTCTGGGTGCAGTAGGTA	
**26**	COMT M40A F	AGAATGGGCCGCGAACGTTGGTG	
**27**	COMT M40K F	GGAATGGGCCAAAAATGTGGGTG	
**28**	COMT M40R F	GGAATGGGCCCGTAATGTGGGTG	
**29**	COMT M40H F	GGAATGGGCCCATAATGTGGGTG	
**30**	COMT M40S F	GGAATGGGCCTCTAATGTGGGTG	
**31**	COMT M40C F	GGAATGGGCCTGCAATGTGGGTG	

Table S1. Table of primers used for gene amplification of CmoA and mutagenesis of CNMT and COMT enzymes. F = forward primer, R = reverse primer.

References

[S1]. G. MacBeath, P. Kast & D. Hilvert, *Biochemistry* **37**, 10062–10073 (1998).

Author Contributions

JM conceived the study; AH, SS, VC and JM designed experiments; AH, VC and SS carried out experiments; MB and RS provide technical assistance; AH, SS, VC and JM analysed data; AH and JM wrote the manuscript
